# Co-managing the double burden: strategies for simultaneously tackling infectious and non-communicable diseases

**DOI:** 10.1186/s40249-026-01477-y

**Published:** 2026-07-31

**Authors:** Jiao-Jiao Li, Mei-Lan Fu, Lingyun-Fei Kong, Zi-Yu Zhao, Okugbe Ebiotubo Ohore, Robert Bergquist, Marcel Tanner, Guo-Jing Yang

**Affiliations:** 1 Key Laboratory of Tropical Translational Medicine of Ministry of Education, School of Public Health, Hainan Academy of Medical Sciences, Hainan Medical University, Haikou, Hainan China; 2 Swiss Tropical and Public Health Institute, Basel, Switzerland; 3 University of Basel, Basel, Switzerland; 4 Ingerod, Brastad, Sweden

**Keywords:** Co-managing, Infectious disease, Non-communicable disease, Association, Hidden link

## Abstract

**Background:**

Evidence increasingly shows close, bidirectional links between infectious diseases (IDs) and non-communicable diseases (NCDs). However, research, health policy, and prevention practices still largely address them in separate frameworks. This scoping review summarises the current evidence on the interconnections between IDs and NCDs and explores integrated strategies for their prevention and management.

**Methods:**

This scoping review systematically searched PubMed, Web of Science, and Scopus for English-language systematic reviews and meta-analyses published between Jan 1, 2000, and April 20, 2026. Two reviewers independently screened eligible studies and extracted effect estimates for associations between IDs and NCDs. Evidence was grouped into two themes: associations between infectious agents and cancer, and the effects of NCDs on susceptibility to or severity of IDs.

**Results:**

From 5799 records identified, 41 meta-analyses or systematic reviews met the inclusion criteria. The included reviews showed associations between pathogen infections and specific cancers, including hepatitis B and C virus infections with liver cancer, human papillomavirus infection with cervical cancer, and *Helicobacter pylori* infection with gastric cancer. IDs were also associated with an increased risk or burden of NCDs, as illustrated by the association between tuberculosis and chronic obstructive pulmonary disease. Conversely, NCDs could affect susceptibility to and outcomes of IDs; patients with diabetes or cardiovascular disease had higher risks of severe disease or adverse outcomes following tuberculosis, influenza, or COVID-19 infection.

**Conclusion:**

IDs and NCDs can interact through shared mechanisms, overlapping risk factors, and bidirectional pathways, underscoring the need for integrated strategies that combine prevention, early detection, treatment, and long-term management. At the public health level, this calls for health policies and financing mechanisms to move beyond disease-specific silos towards systemic frameworks that support integrated prevention and control, coordinated management, and cross-sectoral action.

**Graphical Abstract:**

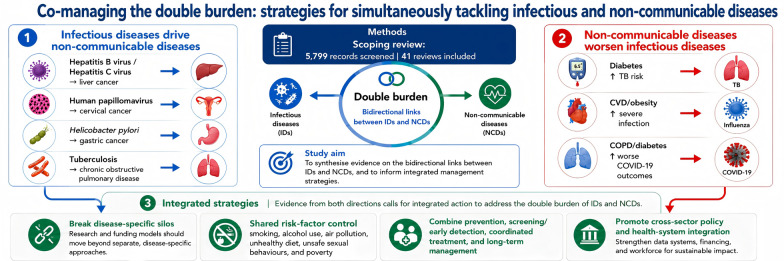

**Supplementary Information:**

The online version contains supplementary material available at 10.1186/s40249-026-01477-y.

## Background

In public health, diseases have traditionally been classified into two broad categories [1]: infectious diseases (IDs) and non-communicable diseases (NCDs). The former are caused by transmissible pathogens, including bacteria, viruses, parasites, and fungi; the latter are typically chronic conditions driven by environmental exposures, lifestyle factors, genetic susceptibility, and their interactions [2]. However, this classification does not imply that the two categories are independent in their occurrence or progression. Infections can contribute to the development of certain NCDs through sustained inflammation, immune dysregulation, metabolic perturbation, and organ injury [3]. In turn, pre-existing NCDs—including diabetes, cardiovascular disease, chronic respiratory disease, and obesity—can increase susceptibility to infection and worsen disease severity and post-infection outcomes [4,5]. Thus, IDs and NCDs should not be viewed as discrete domains, but rather as conditions that interact in complex and persistent ways across pathogenesis, disease progression, and clinical outcomes.

**Fig. 1:**
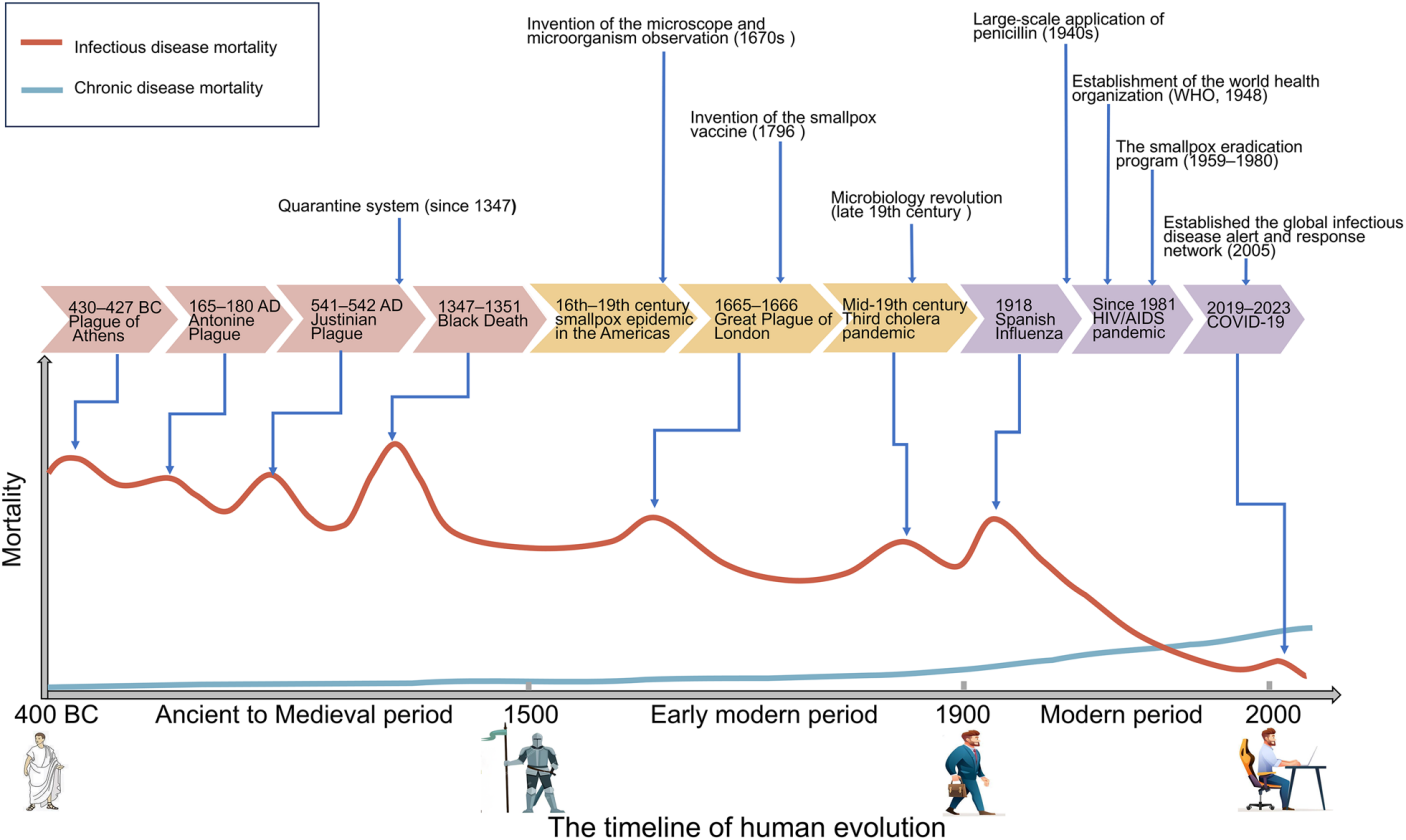
Approximate impact of major historical infectious disease outbreaks on mortality Figure legend: The X-axis represents the time line covering the last 2500 year of human history and the Y-axis represents the concurrent mortality rates. The blue line represents the rising mortality rate due to chronic diseases over time. The dark red line represents the fluctuating mortality rate due to IDs over time. *BC* Before Christ, *AD* Anno Domini, *HIV* Human immunodeficiency virus, *AIDS* Acquired immunodeficiency syndrome, *COVID-19* Coronavirus disease 2019, *WHO* World Health Organization

From the perspective of the epidemiologic transition, the burden of IDs and NCDs has changed dynamically over time [6]. Before the establishment of modern medicine and public health systems, IDs accounted for a substantial share of population morbidity and mortality. With the establishment of germ theory and the development of interventions such as antisepsis, antibiotics, and vaccines, the capacity to prevent and control IDs improved substantially [7]. The approximate impact of major historical IDs outbreaks on mortality is shown in Fig. 1. Meanwhile, NCDs have become an increasingly prominent global health burden amid population ageing and changing lifestyles. World Health Organization (WHO) estimates indicate that, in 2021, NCDs caused 41 million deaths, accounting for 74% of all deaths worldwide, and were among the leading causes of death and disability globally [8]. This epidemiological transition, however, does not signify the end of IDs; on the contrary, emerging and re-emerging infections, including severe acute respiratory syndrome and coronavirus disease 2019 (COVID-19), have appeared in succession, highlighting the persistent threat posed by IDs. The coexistence and interaction of IDs risks with the growing burden of NCDs constitute a double burden that public health systems must urgently address, particularly in low-income and middle-income countries (LMICs) [9].

While there has been growing recognition of the breadth of linkages between IDs and NCDs, the two fields remain largely isolated from each other [10]. They are often supported by separate funding streams, studied using divergent research methodologies, and shaped by vertical health policies and programme frameworks. Such fragmentation can weaken health systems’ ability to identify and respond to shared risk factors, overlapping mechanisms, and compounded disease burdens, while also impeding the efficient allocation of limited health-care resources [11]. An integrated perspective is therefore needed to re-examine the interactions between these two disease categories.

This Review aims to synthesise current evidence on the links between IDs and NCDs, with a focus on their interrelated effects, shared risk factors, and potential pathogenic mechanisms. It also examines the need for integrated management strategies to inform coordinated responses to the double burden of disease.

## Methods

This scoping review aims to map the existing evidence on the bidirectional associations between IDs and NCDs, with particular emphasis on the associations between infectious agents and cancer initiation and progression, and on the influence of NCDs on susceptibility to or clinical outcomes of IDs. The review was conducted according to the methodological framework proposed by Arksey and O’Malley [12] and is reported in accordance with the PRISMA-ScR guidelines [13].

### Search strategy

We searched PubMed, Web of Science, and Scopus for studies published from Jan 1, 2000, to April 20, 2026. The initial search used broad combinations of keywords, including “infectious diseases”“communicable diseases” “non-communicable diseases” “chronic diseases” “association” “interaction” and “comorbidity” to identify major disease pairings relevant to associations between IDs and NCDs. Search strategies were then refined for the main disease pairings on the basis of the preliminary search results and input from clinical experts. Detailed search strategies for each database are provided in supplementary file 1. We also screened the reference lists of included studies and relevant reviews to identify additional potentially eligible studies. Epidemiological and disease burden data were obtained primarily from authoritative sources, including WHO and the Global Burden of Diseases, Injuries, and Risk Factors Study 2021.

### Inclusion and exclusion criteria

Studies were eligible for inclusion if they were published in English, involved human populations or population-level data, examined associations between IDs and NCDs, and were systematic reviews or meta-analyses. For specific disease pairings for which no relevant systematic review or meta-analysis was available, observational studies, including case-control and cohort studies, were considered as supplementary evidence.

Studies were excluded if they did not address associations between IDs and NCDs; were based solely on animal experiments, in vitro experiments, or molecular mechanistic studies without supporting population-level evidence; were non-original or non-systematic publications, such as editorials, commentaries, letters, conference abstracts, or news reports; were duplicate publications; were earlier reviews that substantially overlapped with newer or more comprehensive reviews in terms of the research question and evidence base; or had no accessible full text or insufficient information for evidence extraction.

### Study screening

All records retrieved from the databases were imported into EndNote X9 (Clarivate, Philadelphia, USA), and duplicates were removed. Study screening was conducted in two stages according to the predefined eligibility criteria: titles and abstracts were first screened, followed by full-text review to determine final inclusion. Two reviewers independently screened all records, and discrepancies were resolved by discussion.

### Quality assessment of included literature

We did not undertake a formal critical appraisal of the included studies, as our aim was to map the scope and key characteristics of the evidence rather than to assess the certainty of findings or quantitatively synthesise results, as would be expected in a systematic review.

### Evidence extraction

Using a prespecified data-extraction framework, we systematically extracted disease combinations, effect estimates or epidemiological measures, and corresponding 95% confidence intervals (*CI*s) from the included studies. Extracted measures included odds ratios (*OR*s), risk ratios (*RR*s), hazard ratios, prevalence, incidence, and mortality. Evidence was organised according to the research questions into two categories: associations between infectious agents and specific cancer types, and associations between NCDs and susceptibility to, severity of, and clinical outcomes of IDs. Findings were summarised descriptively and presented in tables. Detailed characteristics of the included studies are presented in Supplementary file 2.

## Results

### Summary of eligible studies

**Fig. 2:**
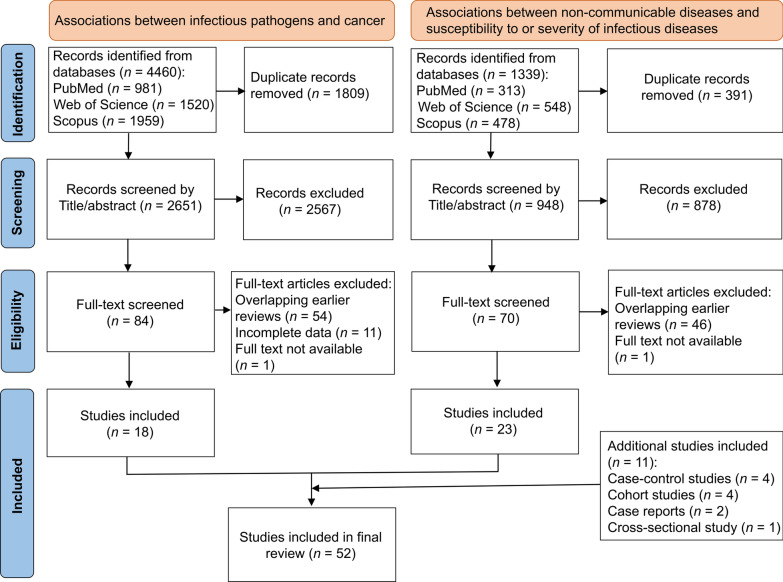
Flow chart of literature search and study selection

Our search across multiple databases identified a total of 5799 potential studies. After removal of duplicates, 3599 records underwent title and abstract screening, of which 3445 were excluded as irrelevant. The full texts of the remaining 154 articles were assessed for eligibility, and 113 were further excluded. In addition, 11 observational studies were identified and included. Overall, 52 eligible studies were included in this review. The study selection and inclusion process is shown in Fig. 2.

Globally, the burden of infection-attributable cancer remains substantial. Infections accounted for an estimated 16.1% of all incident cancer cases in 2008 (2.0 million of 12.7 million cases) [14] and 12.2% in 2018 (2.2 million of 18.1 million cases) [3]. These estimates were based on infectious agents classified by the International Agency for Research on Cancer (IARC) as carcinogenic to humans, including *Helicobacter pylori*; human papillomavirus (HPV), hepatitis B virus (HBV), hepatitis C virus (HCV), Epstein-Barr virus (EBV), human herpesvirus 8, and human T-cell leukemia virus type 1 (HTLV-1); and *Schistosoma* species and liver flukes. HIV was excluded from these estimates because it increases cancer risk indirectly through immunosuppression rather than through direct carcinogenicity.

Since these estimates were published, IARC has classified hepatitis D virus and Merkel cell polyomavirus as Group 1 carcinogens, increasing the number of IARC-recognised carcinogenic infectious agents to 13 [15]. Beyond these established pathogens, accumulating evidence implicates additional infectious agents in carcinogenesis. The study integrates IARC-classified carcinogenic pathogens with evidence from the published literature to summarise associations between infectious agents and cancer sites (Table 1).

**Table 1:** Major infectious pathogens (viruses, bacteria, and parasites) associated with specific cancers: mechanisms, transmission, disease burden, evidence of association, cancer risk factors, prevention strategies, and available WHO 2030 elimination targets

Pathogen	Linked cancer	Pathogenic mechanism	Transmission	Burden of disease	Degree of association	Cancer risk factors	Prevention	Elimination target 2030
Hepatitis B virus (HBV) [36]	Hepatocellular carcinoma (HCC)	Chronic inflammation [37]; interference with p53 tumour suppression [38]; epigenetic modification [39]	Through body fluids; sexual and vertical [40]	World Health Organization (WHO): 254 million chronic hepatitis B cases in 2022, 1.2 million new infections/year [41]	HBV infection, indicated by HBsAg positivity, was associated with an increased risk of HCC (*OR* = 8.90, 95% *CI*: 6.00–13.00) [42]	Genetic disposition; older age; male gender [43]; smoking; metabolic disorder; alcohol abuse [44]	Vaccination [37]; regular screenings; low-risk lifestyle	< 520,000 infected; < 500,000 deaths [45]
Hepatitis C virus (HCV)	HCC	Chronic inflammation [37]; interference with p53 tumour suppression [38]; epigenetic modification [39]	Through blood-contact, sexual and vertical [40]	50 million chronic hepatitis C cases worldwide, 1 million new infections/year [46]	Risk of HCC in patients with HCV (*OR* = 7.70, 95% *CI*: 5.60–10.60) [42]	Genetic disposition; older age; male gender [43]; smoking; metabolic disorder; alcohol abuse [44]	Vaccination [37]; regular screenings; low-risk lifestyle	< 520,000 infected; < 500,000 deaths [45]
	Non-Hodgkin lymphoma (NHL)	Persistent HCV antigen stimulation leads to B-cell proliferation, immune dysfunction, and B-cell malignancies [47]	Unsafe injections; blood-contact sexual practices [46]	NHL had 509,590 new cases and 248,724 deaths worldwide in 2018 [48]	Positive association between HCV infection and NHL (*OR* = 1.69, 95% *CI*: 1.40–2.03) [47]	Cryoglobulin, female sex, long-lasting infection and being from an HCV endemic region [49]	Antiviral medications should be administered as early as possible [50]	No elimination target set
Hepatitis D virus (HDV) (Co-infection with HBV and HDV)	HCC	The intricate mechanisms driving the carcinogenicity of the HDV and HBV are not fully elucidated; involve chronic inflammation, immune dysregulation, and the direct oncogenic effects of the HDV [51]	Injection drug use; percutaneous exposure (e.g., needlestick injuries); sexual exposure [52]	~ 12–72 million people worldwide; ~ 20%–50% of infected individuals diagnosed [52]	HDV infection significantly increases HCC risk (*OR* = 1.28, 95% *CI*: 1.05–1.57) [53] A significantly higher risk of HCC among patients with HDV/HBV dual infection (*OR* = 2.08, 95% *CI*:1.37–3.14) compared with those with HBV monoinfection [54]	Contaminated needles; unsterilized equipment during medical procedures; sharing of contaminated household articles [52]	HBV vaccine and hepatitis B immunoglobulin. Chronic HBV patients: avoid parenteral or sexual contact with HDV-infected individuals [52]	No independent targets; elimination achieved through HBV elimination
Hepatitis E virus (HEV)	HCC	Unclear	Fecal-oral route; principally via contaminated water [55]	~ 19.47 million cases of acute hepatitis and 3450 deaths globally in 2021; responsible for ~ 5.4% of global disability-adjusted life years related to acute hepatitis [55]	HEV infection significantly increases HCC risk (*OR* = 1.94, 95% *CI*: 1.26–3.00) [56]	Drinking contaminated water; consumption of uncooked or undercooked meat [55]	Safe drinking-water; sanitation; food hygiene; vaccination [55]	No elimination target set
Epstein-Barr virus (EBV)	Hodgkin lymphoma (HL)	Anti-apoptotic signals; B-cell receptor loss and chronic inflammation modulate its gene expression and latent membrane protein 1 oncogenicity [57]	Through saliva and other body fluids [58]	There were 78,800–87,600 new cases of HL and 20,100–27,000 resulting deaths in 2020 [21]	EBV-positive Hodgkin's lymphoma risk increased (*RR* = 4.00, 95% *CI*: 3.40–4.50) [59]	Familial factors; viral exposures and immune suppression [60]	Intravenous immunoglobulin; antiviral chemoprophylaxis [61]	No elimination target set
	Burkitt lymphoma	Latent protein activity; Immune evasion; Genomic instability; Microenvironmental factors [62]	Exchange of saliva or contact with the airborne virus [63]	Incidence 2–7 per 1,000,000 person-years in Europe [64]	The prevalence of EBV in patients with Burkitt lymphoma was 57.5% (95% *CI*: 51.50–63.40) [65]	Organ transplantation [66]	Limit close contact with infected individuals; practice safe sex [67]	No elimination target set
	Nasopharyngeal cancer (NPC)	Alteration of host epigenetics: immune evasion; induction of stem cell-like properties [21]	Saliva and other body fluids [58]	By age 40 years, 90%–95% of all adults have been infected [68,69] 200,000–250,000 cases annually [21,70]	EBV detected in about 98% of all NPC tumours [27]	Genetic disposition; consumption of preserved food (due to nitrosamine): common in Southeast Asia [71]	Early detection by serology screening high-risk groups [72]	No elimination target set
	Gastric cancer (GC)	Host genome hypermethylation; p53 pathway evasion; Viral entry via inflammation; Viral gene expression; Mutation profile [73]	Saliva and other body fluids [58]	The global prevalence of EBV in GC to be 8.7% [74]	EBV infection is associated with more than 18 times increase the risk of GC (*OR* = 18.56, 95% *CI*: 15.68–21.97) [75]	Heavy alcohol; history of previous gastric ulcer [76]	Intravenous immunoglobulin; antiviral chemoprophylaxis [61]	No elimination target set
Human papillomavirus (HPV)	Cervical cancer (CC)	Infected basal epithelial cells after microabrasion; genomic integration; immune evasion [77]	Sexual and vertical infections [77]	630 million [78] 690,000 (~ 5% of all cancers globally) [79]	HPV16 is the most strongly associated genotype with invasive cervical cancer (*OR* = 48.3, 95% *CI*: 45.7–50.9) [80]	Early sexual debut; multiple partners; STI co-infections [81–83]	Vaccination; testing and screening [84]	90% of girls vaccinated; 70% screened at ages 35 and 45 years old [85]
	Oropharyngeal carcinoma (OPC)	High-risk HPV binds to cellular chromosomes, with E6 degrading p53 and E7 inactivating Rb, promoting carcinogenesis; immune evasion [19]	Sexual; indirect contact and vertical transmission [77]	Globally, OPC accounts for 76% of approximately 38,000 HPV-related head and neck cancer cases [86]	Salivary HPV-16 positivity associated with OPC (*OR* = 38.50, 95% *CI*: 22.43–66.07) [87]	Early sexual activity; multiple sexual partners; age ≥ 30 [81]	HPV vaccination [84]	90% of girls vaccinated; 70% screened at ages 35 and 45 years old [85]
	Larynx cancer (LC)	EGFR activation and TGF-βR loss together drive proliferation, block apoptosis, and promote cancer [86]	Sexual; indirect contact and vertical transmission [77]	Globally, approximately 4,400 cases of HPV-related LC occurred in 2020 [19]	HPV infection is associated with laryngeal squamous cell carcinoma (*OR* = 5.39, 95% *CI:* 3.25–8.94) [88]	Smoking, alcohol consumption, and EBV infection [86]	HPV vaccination [19]	90% of girls vaccinated; 70% screened at ages 35 and 45 years old [85]
	Anal cancer (AC)	HPV16 E6/E7 degrades p53 and inactivates retinoblastoma protein, disrupting DNA repair and cell cycle, driving anal intraepithelial neoplasia to invasive cancer [89]	Sexual; indirect contact and vertical transmission [77]	AC cases rose by an average of 2.2% annually over the past decade, accounting for 0.5% of all new cancer cases in 2024 [90]	Anti-HPV58 E6 antibodies were associated with anal cancer (*OR* = 6.80, 95% *CI*: 1.40–33.10) [91]	Smoking and sexual practices such as history of anal receptive intercourse [92]	HPV vaccination [89]	90% of girls vaccinated; 70% screened at ages 35 and 45 years old [85]
	Penis carcinoma (PeC)	HPV integration; E6 (↓p53) and E7 (↓Rb);cell cycle dysregulation; p16 overexpression; epigenetic changes; immune evasion [93]	Sexual; indirect contact and vertical transmission [77]	The total number of PeC globally has been estimated to be 26,000 cases per year [94]	Over 50% of the PeC are attributable to HPV, and of these, 73% caused by the HPV 16/18 strains [95]	Phimosis, smoking, and low social economic status; age > 60 years [96]	HPV vaccination; safe sex [93]	90% of girls vaccinated; 70% screened at ages 35 and 45 years old [85]
	Vagina carcinoma (VaC)	Persistent infection; vulvar and vaginal intraepithelial neoplasia [97]	Sexual; indirect contact and vertical transmission [77]	VaC accounts for approximately 17,908 new cases annually worldwide [98]	In a cohort of 79 VaC, HPV DNA was detected in 98.6% of squamous cell carcinomas, HPV‑positivity was associated with improved survival (*HR* = 0.14) [99]	Smoking; having multiple sexual partners; engaging in sexual activity at an early age [100]	ISA101 vaccination [101]	90% of girls vaccinated; 70% screened at ages 35 and 45 years old [85]
	Vulva carcinoma (VC)	HPV integration disrupts E2, leading to E6/E7 expression and carcinogenesis [102]	Sexual; indirect contact and vertical transmission [77]	VC is rare, with approximately 45,000 new cases worldwide annually [103]	The prevalence of HPV in VC in case–control studies was 30% (*OR* = 10.46) [104]	Smoking and immunosuppression [105]	HPV vaccination [106]	90% of girls vaccinated; 70% screened at ages 35 and 45 years old [85]
Kaposi sarcoma herpesvirus(KSHV)	Kaposi sarcoma (KS)	Latent infection hijacks NF-κB, PI3K/Akt, and JAK/STAT pathways, evades immunity, and induces inflammation [107]	Sexual contact; saliva transmission [108]	There were roughly 42,000 new KS cases and 20,000 deaths estimated in 2018 worldwide [109]	KSHV seropositivity is strongly associated with KS (*OR* = 9.6, 95% *CI*: 2.90–31.50) [110]	High-sugar diet, coronary heart disease, genetic variations in HLA-DQB1/DRB1 [111]	Antiviral and other prevention strategies remain exploratory [112]	No elimination target set
Human T-cell lymphotropic virus (HTLV)	Adult T-cell leukemia and lymphoma (ATLL)	HTLV-1 causes ATL via persistent T-cell infection, clonal expansion, and immune evasion, driving mitotic mutations [113]	Sexual transmission; vertical transmission; contaminated blood products [114]	HTLV-1 is a Deltaretrovirus that infects 10–20 million people worldwide [115]	The estimated lifetime risks of ATLL in HTLV-1 carriers is 3%–5% [116]	Higher proviral load, advanced age, family history of ATL [117]	Blood screening, partner testing and condom promotion [118]	No elimination target set
Human cytomegalovirus (HCMV)	Childhood Acute Lymphoblastic Leukemia (ALL)	Induces chromosomal instability; disrupts host immune balance; oncomodulatory [119]	Through bodily fluids; vertical transmission [119]	HCMV infection has a 0.64% global prevalence and a 17%–20% risk of serious long-term effects in children [120]	In utero infection with HCMV is a risk factor for ALL (*OR* = 3.71, 95% *CI*: 1.56–7.92, *P* = 0.0016) [119]	HCMV exposure during pregnancy or early life[121]	Universal hygiene education; establishing HCMV serological status [119]	No elimination target set
Merkel cell polyomavirus (MCPyV)	Merkel cell carcinoma (MCC)	Persistent MCPyV infection—LT truncation mutation—cell proliferation [122]	Skin contact [123]	MCC is a highly lethal skin cancer with a mortality rate approaching 50% and its incidence has tripled over the past two decades [122]	Compared with the control group, MCPyV infection was significantly associated with MCC (*OR* = 3.51, 95% *CI*: 2.96–4.05) [124]	Excessive ultraviolet exposure; advanced age; immunosuppression [122]	Reduce exposure to ultraviolet rays [125]	No elimination target set
*Schistosoma haematobium*	Bladder cancer (BC)	Chronic inflammation; triggered by egg immune responses in bladder wall; anti-apoptosis; gene mutation [126,127]	Exposure to infected snail host in water[126]	112 million people infected with schistosomiasis in sub-Saharan Africa [128]. BC: definite figure unknown but estimated at > 20,000 [129]	History of schistosomiasis increased the risk of BC 1.4‑fold in men (*OR* = 1.40, 95% *CI*: 1.20–1.70) and 1.9‑fold in women (*OR* = 1.90, 95% *CI*: 1.20–3.00) [130]	Chronic infection; genetic disposition [126,127]	MDA + snail control; sanitation; health education [131]	Elimination to be achieved globally by 2030 [132]
*Schistosoma japonicum*	Colorectal cancer (CRC)	Chronic inflammation; egg immune responses in intestines; anti-apoptosis; gene mutation [133]	Exposure to freshwater containing cercariae from infected *Oncomelania* snail [134]	*S*. *japonicum* infection in Asia: ~ 100 million people infected [135]. A bout a few thousand cases annually [136]	The study found a significant association between previous schistosomal infection and colon cancer, with an OR of 3.3 (*OR* = 3.30, 95% *CI*:1.80–6.10) [137]	6.3%–37.1% CRC prevalence in patients with chronic infection [138]	MDA + snail control; sanitation; health education [139]	Elimination to be achieved globally by 2030 [132]
*Clonorchis sinensis*	Cholangiocarcinoma (CCA)	Chronic inflammation, oxidative/nitrative stress, nitrosamine production, abnormal cell proliferation, and epigenetic alterations, leading to carcinogenesis [140]	Raw or not fully cooked, infected freshwater fish [141]	*C. sinensis*:15 to 20 million [142]	Associated with increased odds of cholangiocarcinoma (*OR* = 4.49, 95% *CI*: 3.43–5.87) [25]	Genetic susceptibility; other biliary tract diseases [143]	Health education; extensive, repeated stool examinations [144]	Morbidity control ; reduction by 75% of those requiring intervention [145]
*Opisthorchis viverrini *	CCA	Induction of cell growth; and anti-apoptosis by parasite excretions bile ducts, changes in biliary tract microbiome [144]	Raw or not fully cooked, infected freshwater fish [141]	*O. viverrini*: 10 millional[146]	3.69-fold higher odds of cholangiocarcinoma in individuals with O. viverrini infection (*OR* = 3.69, 95% *CI*: 2.07–6.55) [25]	Genetic susceptibility; smoking and drinking; other biliary tract diseases [143,147]	Health education; extensive, repeated stool examinations [144]	Morbidity control ; reduction by 75% of those requiring intervention [145]
Cutaneous Leishmaniasis (CL)	cutaneous squamous cell carcinoma (cSCC)	chronic infection, chronic inflammation, metabolic oxidative stress, apoptosis inhibition, inhibition of tumor suppressors, wound healing, genetic transfer, nonspecific tropism, Leishmania -driven lymphoproliferation, and leishmaniasis-related scarring [148]	The bite of a female sand-fly [149]	From 1990 to 2021, the Disability-Adjusted Life Year (DALY) rate of cutaneous and mucocutaneous leishmaniasis increased from 3.86 to 4.88 per 100,000 (up 26.4%) [150]	In most cases, cSCC developed on the scarring tissue of cutaneous leishmaniasis years later[151]	Previous trauma, Scarring, Unhealed burns, or chronic wounds [151]	Vector control; reservoir control; health education; early detection.[152]	The goal of the WHO 2021–2030 Neglected Tropical Diseases road map is to reduce mortality caused by the disease to less than 1%[153]
	Basal cell carcinoma (BCC)	Same as above [148]	The bite of a female sand-fly [149]	An estimated 700,000 to 1 million new cases of CL occur annually worldwide [151]	Leishmaniasis scar is a recognized risk factor for BCC, with reported cases developing 3 to 59 years after the primary lesion [154]	Male gender; Upper-middle-age; History of malignancies; Previous X-ray irradiation; Leishmanization with the leishmanial scar on the site of BCC tumor [154]	Educate patients on signs of malignancy and emphasize strict sun avoidance [155]	Same as above [153]
Visceral leishmaniasis(VL)	Lymphoma	Chronic infection, chronic inflammation, metabolic oxidative stress, apoptosis inhibition, inhibition of tumor suppressors, wound healing, genetic transfer, nonspecific tropism, Leishmania -driven lymphoproliferation, and leishmaniasis-related scarring [148]	The bite of a female sand-fly [149]	In 2021, the age- standardized DALY rate for VL was 5.39 per 100,000 population (95% *UI*: 1.70–17.12) [156]	Coexistence of lymphoma and leishmaniasis in the same node [157]	Aging; ultraviolet radiation; abuse of animal saliva; herbal recipes; corrosive chemicals and topical steroids [148]	Vector control and reservoir control [158]	The goal of the WHO 2021–2030 Neglected Tropical Diseases road map is to reduce mortality caused by the disease to less than 1% [153]
*Cryptosporidium*	*Colon cancer*	Chronic inflammation; manipulation of the immune system; alteration of cell signaling pathways [159]	The fecal-oral route [160]	Top cause of moderate-to-severe diarrhea in young children, ranking fourth globally among diarrheal pathogens [159]	Cryptosporidium infection increases the risk of colorectal cancer by approximately 19 times (*OR* = 19.12, 95% *CI*: 4.82–75.99) [161]	Household diarrhoea, poor quality drinking water, animal contact, open defecation/lack of toilet [162]	Improvements in water, sanitation, and hygiene (WASH); protecting high-risk populations [163]	No elimination target set
*Helicobacter pylori*	Gastric cancer (GC)	Chronic subclinical inflammation of gastric epithelium [146]	Oral-to-oral; faecal-oral transmission [164]	During 2015–2022, the global prevalence of *H. pylori* in adults was 43.9% [165]	*H. pylori *infection was strongly associated with GC in both Western and Asian populations, with a larger effect size in Europe/North America (*OR* = 5.37) than in Asia (*OR* = 2.50) [28]	High alcohol intake; consumption of preserved food (due to nitrosamine) [164]	Non-consumption of preserved food; controlled alcohol intake; *H. pylori* eradication [166]	No elimination target set
*Chlamydia pneumoniae*	Lung cancer (LC)	Chronic sub-clinical inflammation (suspected but not definitively proven) [167]	Airborne transmission [32]	~ 2.5 million new cases annul [168]	*C. pneumoniae* infection is associated with increased risk of LC (*OR* = 1.48, 95% *CI*: 1.32–1.67) [32]	Polluted air; smoking (incl. second hand); family cancer history [169]	Quit smoking; early screening [170]	No elimination target set

*HBV* Hepatitis B virus, *HCC* Hepatocellular carcinoma, *WHO* World Health Organization, *HBsAg* hepatitis B surface antigen, *OR* odds ratio, *CI* confidence interval, *HCV* Hepatitis C virus, *NHL* Non-Hodgkin lymphoma, *HDV* Hepatitis D virus, *HEV* Hepatitis E virus, *EBV* Epstein-Barr virus, *HL* Hodgkin lymphoma, *RR* risk ratio, *NPC* Nasopharyngeal cancer, *GC* Gastric cancer, *HPV* Human papillomavirus, *CC* Cervical cancer, *STI* sexually transmitted infection, *OPC* Oropharyngeal carcinoma, *LC* Larynx cancer, *EGFR* epidermal growth factor receptor, *TGF-βR* transforming growth factor beta receptor, *AC* anal cancer, *DNA* deoxyribonucleic acid, *PeC* Penis carcinoma, *VaC* Vagina carcinoma, *HR* hazard ratio, *VC* vulva carcinoma, *KSHV* Kaposi sarcoma herpesvirus, *KS* Kaposi sarcoma, *NF-κB* nuclear factor kappa B, *PI3K/Akt* phosphoinositide 3-kinase/protein kinase B, *JAK/STAT* Janus kinase/signal transducer and activator of transcription, *HLA-DQB1* human leukocyte antigen DQB1, *HTLV-1* human T-cell lymphotropic virus, *ATLL* Adult T-cell leukemia and lymphoma, *HTLV-1* human T-lymphotropic virus type 1, *ATL* adult T-cell leukemia, *HCMV* Human cytomegalovirus, *ALL* acute lymphoblastic leukemia, *MCPyV* Merkel cell polyomavirus, *MCC* Merkel cell carcinoma, *LT* large T antigen, S. haematobium Schistosoma haematobium, *BC* bladder cancer, *MDA* mass drug administration, *S*. japonicum Schistosoma japonicum, *CRC* Colorectal cancer, *C.* sinensis, Clonorchis sinensis, *CCA* cholangiocarcinoma, *O*. viverrini Opisthorchis viverrini, *CL* Cutaneous leishmaniasis, *cSCC* cutaneous squamous cell carcinoma, *DALY* disability-adjusted life year, *BCC* Basal cell carcinoma, *VI* visceral leishmaniasis, *UI* uncertainty interval, WASH water sanitation and hygiene, *H*. pylori Helicobacter pylori, *C*. pneumoniae Chlamydia pneumoniae, *IgA* immunoglobulin A, *IgG* immunoglobulin G, *HLA-DRB1* human leukocyte antigen DRB1

### Viral infections associated with cancer

HBV and HCV are the major infectious causes of hepatocellular carcinoma (HCC) [3,16] The risk of HCC is 12.5-fold higher among individuals infected with HBV and 11.2-fold higher among those infected with HCV than among uninfected individuals [17]. In 2022, the global attributable fractions of HCC due to HBV and HCV were 52% and 21%, respectively [18].

HPV is associated with several anogenital cancers including cancers of the cervix, vagina, vulva, penis, and anus, and is also commonly implicated in oropharyngeal carcinoma [19]. Persistent infection with high-risk HPV types is estimated to cause 90% of cervical and anal cancers, 70% of vulvar and vaginal cancers, 60% of penile cancers, and 70% of oropharyngeal cancers [20].

EBV has an important role in the development of nasopharyngeal carcinoma (NPC). High-incidence regions include Eastern and Southeastern Asia and parts of the Middle East, where EBV is associated with more than 95% of NPC cases. In regions with lower NPC incidence, the proportion of EBV-associated cases is lower, at approximately 75% [21].

### Parasitic infections associated with cancer

Chronic infection with *Schistosoma haematobium* is causally associated with bladder cancer, with sufficient evidence supporting this association, and has been classified by IARC as a Group 1 carcinogen. Epidemiological studies have shown that *S. haematobium* infection increases the risk of bladder cancer by approximately 2–15-fold [22]. Pathological studies from highly endemic regions have detected schistosome eggs in 82.4% of bladder cancer specimens [23]; in parts of Africa, up to 85% of bladder squamous cell carcinomas are thought to be attributable to chronic infection [24].

*Opisthorchis viverrini * and *Clonorchis sinensis * are established human carcinogenic parasites, and chronic infection with these liver flukes is closely associated with cholangiocarcinoma. A recent systematic review and meta-analysis showed a significant association between liver fluke infection and cholangiocarcinoma, with infected individuals having approximately 4.24-fold higher odds of developing cholangiocarcinoma than uninfected individuals. The corresponding estimates were 4.49-fold for *C. sinensis* infection and 3.69-fold for *O. viverrini* infection [25].

### Bacterial infections associated with cancer

The global prevalence of *H. pylori* infection remains high, with an estimated prevalence of 43.1% between 2011 and 2022 [26]. Although most infected individuals do not develop gastric cancer, approximately 1–3% of those infected in low-risk regions ultimately develop the disease, with higher proportions reported in high-risk regions [27]. *H. pylori* infection substantially increases the risk of non-cardia gastric cancer, by approximately 2.5-fold in Asian populations and 5.4-fold in European and North American populations [28]. The most recent meta-analysis showed that, among infected individuals without gastric neoplasia, eradication therapy was associated with an estimated 36% relative reduction in gastric cancer incidence and a reduction in gastric cancer mortality [29].

Epidemiological studies suggest that previous or chronic infection with *Chlamydia pneumoniae * might be associated with lung cancer development, although its independent carcinogenic role remains controversial [30]. A recent meta-analysis showed that *C. pneumoniae*-specific immunoglobulin A seropositivity was associated with an increased risk of lung cancer, with a pooled *OR* of 3.19; immunoglobulin G seropositivity was also associated with increased risk, with a pooled *OR* of 2.02 [31]. Further prospective studies are needed to validate this association [32].

### Interplay between IDs and NCDs

IDs and NCDs are linked by bidirectional interactions. Chronic NCDs, including diabetes, chronic obstructive pulmonary disease, and cardiovascular disease, substantially increase susceptibility to infection and the risk of adverse outcomes after infection. Conversely, infections can worsen pre-existing chronic diseases and precipitate acute clinical events (Table 2).

**Table 2:** Non-communicable diseases associated with increased susceptibility to or severity of infectious diseases: epidemiology, pathogenic mechanisms, association strength, and preventive strategies

Non-communicable disease (NCDs)	Disease	Associated infectious disease (IDs)	Epidemiology	Pathogenic mechanism	Degree of association	Prevention
Metabolic disease (MD)	Type-2 diabetes mellitus (T2DM)	COVID-19 (SARS-CoV-2 infection)	The overall prevalence of new-onset diabetes post-COVID-19 was 1.37%, with T2DM accounting for 0.84% [171]	Infection improves affinity of viral cellular binding; decreased viral clearance; diminished T cell functions; increased risk for cytokine storm syndrom e [172]	SARS-CoV-2 infection in people living with diabetes was associated with significantly increased mortality (*OR* = 2.52, 95% *CI*: 1.45–4.36, *I *^*2*^ = 99%) [173] COVID-19 infection was associated with an increased risk of new-onset diabetes (*HR* = 1.46, 95% *CI*: 1.38–1.55), including type 2 diabetes (*HR* = 1.47, 95% *CI*: 1.36–1.59) [174] T2DM was significantly associated with higher mortality and greater disease severity in patients with COVID-19 (mortality: *OR* = 3.66, 95% *CI*: 2.20–5.11, *P* < 0.001; severity: *OR* = 1.97, 95% *CI*: 1.02–2.92, *P* < 0.001) [175]	Isolation; use of facemask; social distance; COVID-19 vaccination; health education [176]
		Tuberculosis (TB)	The prevalence of T2DM among TB patients in various low- and middle-income countries (LMICs) ranged from 1.8% to 45%, while the prevalence of TB among people with DM ranged from 0.1% to 6.0% [177] The pooled prevalence of diabetes mellitus among tuberculosis patients in sub-Saharan Africa was 9.0% (95% *CI*: 6.00–12.00) [178]	Diabetes raises the risk of pulmonary tuberculosis (PTB) by disrupting immunity, while PTB impair glucose homeostasis and complicate TB drug therapy [179]	Mendelian randomization studies show that type 2 diabetes causally increases tuberculosis risk (pooled *OR* = 1.22, 95% *CI*: 1.11–1.33) [180] People with diabetes have a 1.5-fold increased risk of developing active TB compared with those without diabetes mellitus (95% *CI*: 1.28–1.76) [181]	Control of blood sugar; continuation of TB drug therapy monitoring potential resistance development [182]
Respiratory disease (RD)	Chronic obstructive pulmonary disease (COPD)	COVID-19	COPD prevalence rates among patients with COVID-19 range from zero to 32.9%[183]	Expression of angiotensin- converting enzyme 2 (ACE2) Vascular constriction and vascular damage[184]	COPD was associated with a higher risk of death among COVID-19 pneumonia patients in Europe (*OR* = 1.84, 95% *CI*: 1.29–2.62), America (*OR* = 1.94, 95% *CI*: 1.50–2.52), and Asia (*OR* = 4.35, 95% *CI*: 2.39–7.62) [185] Compared with those without COPD, patients with COPD had higher risks of COVID-19-related hospitalization (*OR* = 1.45, 95% *CI*: 1.30–1.61), ICU admission (*OR* = 1.28, 95% *CI*: 1.08–1.51), and mortality (*OR* = 1.41, 95% *CI*: 1.37–1.65) [183] Studies conducted in Asia showed a statistically significant difference in COPD incidence between severe and non-severe COVID-19 patients (*OR* = 4.04, 95% *CI*: 3.05–5.34, *I*^2^ = 1%; *P* < 0.00001) [186]	COVID-19 vaccination [187]
		TB	The pooled prevalence of COPD among TB patients was 15.95% (95% *CI*: 11.61–21.53), while the pooled prevalence of TB among COPD patients was 5.57% (95% *CI*: 2.24–13.18) [188]	Small airway involvement; Bronchodilators; Accelerated parenchymal destruction [189]	Pooled analysis shows significant risk elevation of COPD in those with history of TB (*OR* = 2.59, 95% *CI*: 2.12–3.15) [190] Meta-analysis revealed that individuals with a history of TB had a significantly increased risk of developing COPD (pooled *OR* = 2.46, 95% *CI*: 1.95–3.10). Similarly, COPD patients had a significantly elevated risk of developing TB (pooled *OR* = 2.21, 95% *CI*: 1.57–3.11) [188]	Bacillus Calmette-Guérin (BCG) vaccination at early age [190] Treatment with *β*-adrenergic receptor agonists,; bronchodilators [191]; antibiotica and vitamin D Stop smoking and reduced exposure to biomass fuels [192]
		Flu	Seasonal flu causes approximately 291,000 to 646,000 respiratory deaths worldwide each year [193]	Worsens airway inflammatory reactions via activating NLRP3 inflammasome signal transduction [194]	Compared with the non-infected group, the risk ratio for acute exacerbations of COPD in the flu-infected group was 1.34 (95% *CI:* 1.25–1.44) [195]	Vaccination; quitting smoking [193]
Cardiovascular diseases (CVDs)	Coronary artery disease (CAD)	COVID-19	The pooled prevalence of CAD among COVID-19 patients was 14.4% (95% *CI*: 12.7–16.2) [196]	Affection of ACE2 pathways; elevated levels of pro- inflammatory cytokines; hypoxemia; electrolyte imbalance [197]	CAD patients infected with COVID-19 have a higher risk of CVD events (*OR* = 2.24, 95% *CI*: 1.38–3.61) [198] Participants with concurrent CAD at the time of hospital admission had 2.64 times the odds of COVID-19 mortality (*OR* = 2.64, 95% *CI*: 2.30–3.04; *I*^2^ = 45%, *P* < 0.01) [196] CAD was also associated with increased severity of COVID-19 disease (10.8% vs. 5.6%, respectively, for severe vs. non-severe groups; *OR* = 2.28, 95% *CI*: 1.59–3.27; *I*^2^ = 72%; *P* < 0.001) [199] COVID-19 patients with preexisting CAD had a two-fold risk of short-term mortality (*OR* = 2.61, 95% *CI*: 2.10–3.24, *P* < 0.001, *I*^2^ = 73.6%); this risk was higher among Asian cohorts (*OR* = 2.66, 95% *CI*: 1.79–3.90, *P* < 0.001, *I*^2^= 77.3%) compared with European (OR = 2.44, 95% *CI*: 1.90–3.14, *P* < 0.001, *I*^2^: 56.9%) and American (*OR* = 1.86, 95% *CI*: 1.41–2.44, *P* < 0.001, *I*^2^= 0%) populations [200]	COVID-19 vaccination [197]
	Acute myocardial infarction (AMI)	Flu	Relative incidence of AMI was 6.16-fold higher in the first 7 days after flu infection [201]	Endothelial dysfunction Platelet and coagulation activation; Systemic inflammation, particularly affecting atherosclerotic plaques [202]	Flu is associated with a significantly increased risk of acute myocardial infarction (pooled adjusted *OR* = 2.70; 95% *CI*: 1.28–5.72) [203] Influenza infection was associated with an elevated risk of acute myocardial infarction (pooled incidence rate ratio = 4.01, 95% *CI*: 2.66–6.05) [35] There was moderate-certainty evidence that influenza triggers AMI (incidence rate ratio = 5.37; 95% *CI:* 3.48–8.28; *I*^*2*^ = 69.4%) [204]	Influenza vaccination [205]
		COVID-19	Among adult COVID-19 patients, the most common complication was acute myocardial injury (AMI) (incidence 19.38%, 95% *CI*: 13.62–26.81; *I*^*2*^ = 97.5%, *P* < 0.001) [206]	Inflammatory response and plaque instability; Endothelial injury/endotheliitis [207]	Higher in-hospital mortality was observed among patients with both STEMI and SARS-CoV-2 (*OR* = 5.24; 95% *CI*: 3.63–7.56) [203] COVID-19 patients showed an increased risk of incident AMI (*HR* = 1.93, 95% *CI*: 1.65–2.26, *P* < 0.0001, *I*^*2*^ = 83.5%) [208]	COVID-19 vaccination

*NCDs* non-communicable diseases, *IDs* infectious diseases, *MD* metabolic disease, *T2DM* type 2 diabetes mellitus, *SARS-CoV-2* severe acute respiratory syndrome coronavirus 2, *COVID-19* coronavirus disease 2019, *OR* odds ratio, *CI* confidence interval, *I*^2^ inconsistency index, *HR* hazard ratio, *TB* tuberculosis, *LMICs* low-income and middle-income countries, *DM* diabetes mellitus, *PTB* pulmonary tuberculosis, *RD* respiratory disease, *COPD* chronic obstructive pulmonary disease, *ACE2* angiotensin-converting enzyme 2, *ICU* intensive care unit, *BCG* Bacillus Calmette-Guérin, *Flu* influenza, *NLRP3* NLR family pyrin domain-containing 3, *CVDs* cardiovascular diseases, *CAD* Coronary artery disease, *AMI* acute myocardial infarction, *STEMI* ST-segment elevation myocardial infarction

People with diabetes have an approximately 1.5–4-fold higher risk of infection than those without diabetes, and diabetes is consistently associated with more severe outcomes after severe acute respiratory syndrome coronavirus 2 (SARS-CoV-2) infection [33]. Cardiovascular disease also amplifies the burden of infection-related illness: during the Omicron era, pre-existing heart disease was associated with an approximately 27% higher risk of hospital admission after COVID-19, whereas heart failure was associated with an approximately 78% higher risk of death. At the same time, infection itself is an important trigger of cardiovascular events [34]. For example, the risk of acute myocardial infarction increases to about four times the baseline level within 1 month after influenza infection; tuberculosis and SARS-CoV-2 infection have also been associated with an increased risk of subsequent cardiovascular events [35].

## Discussion

IDs and NCDs have traditionally been addressed separately, an approach that has long guided health-care strategies, policies, and research. Mounting evidence from science and experience clearly shows that these two categories are not isolated from one another but are, in fact, deeply intertwined. This bidirectional relationship, together with their shared susceptibility to socioeconomic, cultural, behavioural, and environmental risk factors, is driving a shift towards more integrated health-care strategies. Through analyses of key examples (Tables 1 and 2), this paper reveals the multiple pathways through which IDs and NCDs interact. These interactions jointly affect individual and population health, underscoring the importance of integrated interventions and global public health strategies. We propose five thrusts of further research and analyses to explore the interconnectivity of IDs and NCDs for the benefit of individual and population health using relevant examples.

### IDs as precursors of NCDs

One of the most well-documented links between IDs and NCDs is the role that chronic infections play in the development of certain cancers. HBV and HCV are well established contributors to liver cancer worldwide, particularly in regions where these infections are endemic [209]. Chronic inflammation induced by these viral infections creates a pro-oncogenic environment that promotes cellular mutations and can ultimately lead to hepatocellular carcinoma [37]. Similarly, HPV is a major risk factor for cervical cancer, and its role in triggering cancerous changes in epithelial cells has been extensively studied [210]. Schistosomiasis is another notable example among parasitic infections, particularly in relation to bladder cancer in sub-Saharan Africa and colorectal cancer in East Asia [133,211]. Visceral leishmaniasis (VL) has also been reported to be associated with hematological malignancies, particularly lymphoma [148]. In endemic regions, VL–*Plasmodium* coinfection may further affect host immune responses and clinical outcomes, thereby complicating diagnosis and management [212]. Beyond these examples, chronic conditions, such as peptic ulcers and gastric cancer have been strongly associated with chronic *H. pylori infection* [213]. This bacterium induces long-term inflammation of the gastric mucosa, increasing the risk of ulceration and malignant transformation over time [214]. These examples illustrate that infectious agents are often more than acute health threats; they can also initiate or contribute to long-term chronic disease processes.

The principal mechanisms linking chronic infection to malignancy are largely convergent. Failure to clear pathogens allows persistent infection to establish a chronic inflammatory and immunosuppressive microenvironment that favours malignant transformation [215,216]. Several processes contribute to this mechanistic link between chronic infection and cancer: pathogen-mediated immune evasion (e.g., EBV latency; HPV E6/E7-mediated inactivation of p53 and retinoblastoma protein) [217,218], chronic inflammation characterized by sustained pro-inflammatory signaling (e.g., interleukin-6- and tumour necrosis factor-α-mediated activation of nuclear factor kappa B and signal transducer and activator of transcription 3 in HBV and HCV infection) [219], and reactive oxygen species/reactive nitrogen species-induced deoxyribonucleic acid (DNA) damage and genomic instability (e.g., *H. pylori*-associated oxidative stress) together contribute to the mechanistic basis linking chronic infection to cancer [220].

### NCDs increase vulnerability to IDs

NCDs can increase susceptibility to IDs and exacerbate their severity. Type 2 diabetes mellitus (T2DM) is a prime example of this relationship, as chronic hyperglycaemia in patients with T2DM weakens immune defence by impairing both innate and adaptive immune responses [221]. Studies have shown that people with diabetes are at substantially increased risk of IDs, including tuberculosis, influenza, and pneumonia [222,223]. Looking specifically at COVID-19, analyses have shown that diabetes has been identified as a major risk factor for severe outcomes and higher mortality rates [224]. Poor glycaemic control can exacerbate systemic inflammatory responses [225], leading to more severe complications, including acute respiratory distress syndrome and multiorgan failure [226,227].

Cardiovascular diseases (CVDs) can also complicate the clinical course of IDs. For example, individuals with pre-existing heart conditions may find it harder to cope with the physiological stress induced by infections like influenza or COVID-19 [228,229]. The additional strain on the cardiovascular system can contribute to adverse outcomes, including myocardial infarction and stroke [230]. This synergistic effect underscores the importance of managing NCDs not only to improve quality of life, but also to reduce the risk of severe myocardial infarction and stroke.

**Fig. 3:**
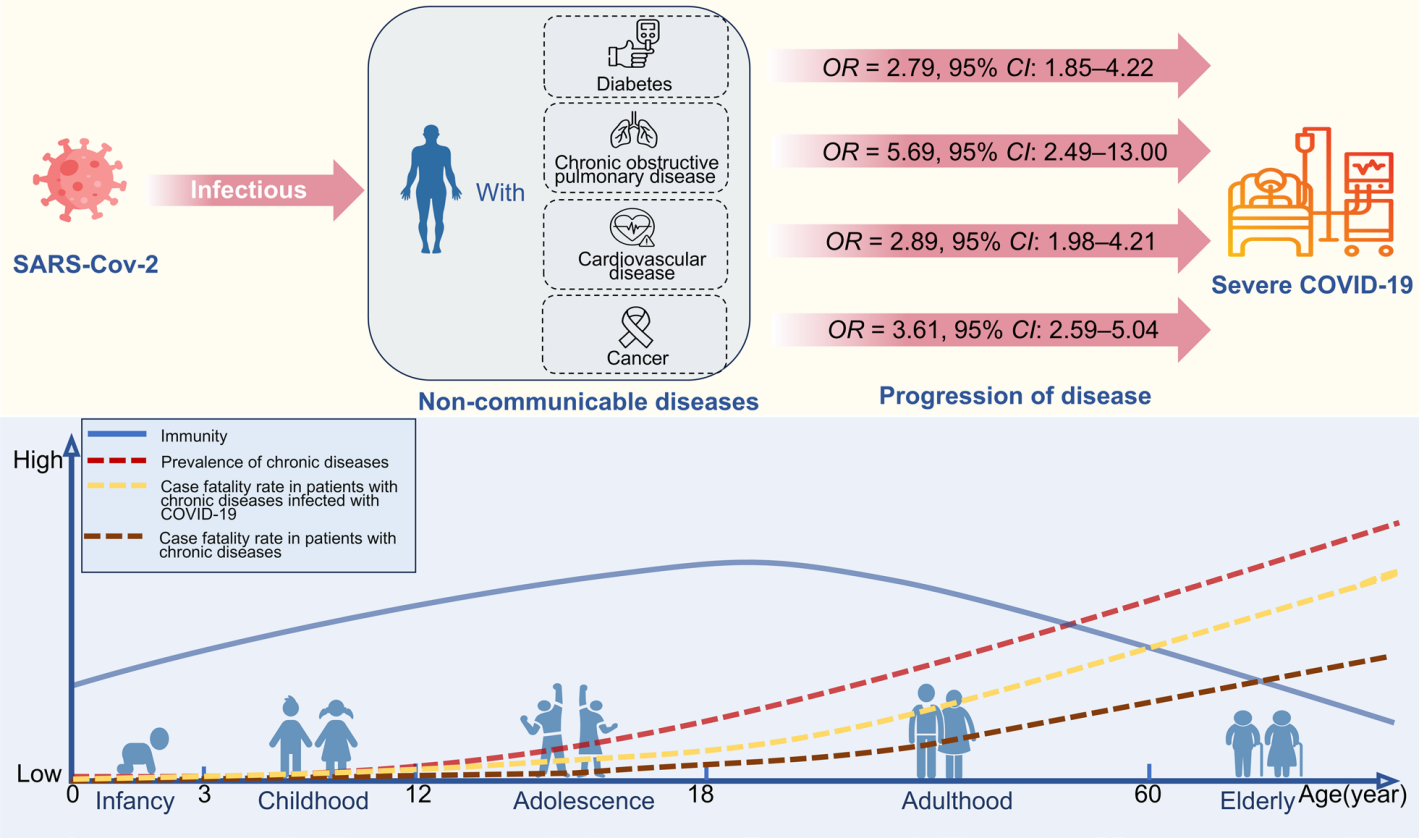
Case illustration: Dynamic interaction between infection, immunity, prevalence and mortality in relation to non-communicable diseases. The upper section shows the odds ratios (*ORs*) and 95% confidence intervals (*CIs*) for accelerated disease progression after SARS-CoV-2 infection in patients with selected pre-existing chronic diseases [32,164,208,209]. The lower section shows temporal changes in immunity (solid blue line), prevalence of chronic diseases (dashed red line), case fatality rate in patients with chronic diseases infected with COVID-19 (dashed yellow line), and case fatality rate in patients with chronic diseases (dashed brown line). Abbreviations: *SARS-CoV-2*, severe acute respiratory syndrome coronavirus 2; *OR* odds ratio, *CI* confidence interval

In addition, patients with chronic obstructive pulmonary disease (COPD) and cancer often experience more severe outcomes after IDs. Studies indicate that patients with COPD are more than five times as likely to develop severe COVID-19 as those without COPD [231]. Similarly, patients with cancer have a more severe clinical course after COVID-19 infection, with a 3.6-fold higher risk of severe COVID-19 than individuals without comorbidities [232] (Fig. 3).

The severity and adverse outcomes of IDs are closely linked to chronic conditions, with immune function having a significant role in this relationship. Immune competence changes dynamically across the life course: it matures from childhood through adolescence, generally reaches its functional peak in early adulthood, and progressively declines with advancing age [233]. In parallel, the burden of chronic diseases increases with age, with a particularly steep rise among older adults [234]. Many chronic conditions are associated with impaired immune responses, which can further compromise host defence against infection. Consequently, patients with chronic diseases who acquire SARS-CoV-2 infection have substantially higher mortality than those without chronic comorbidities [224]. These findings highlight the compounded effect of age-related immune decline and chronic disease on the prognosis of IDs, as illustrated in Fig. 3.

### The epidemiological triangle

**Fig. 4:**
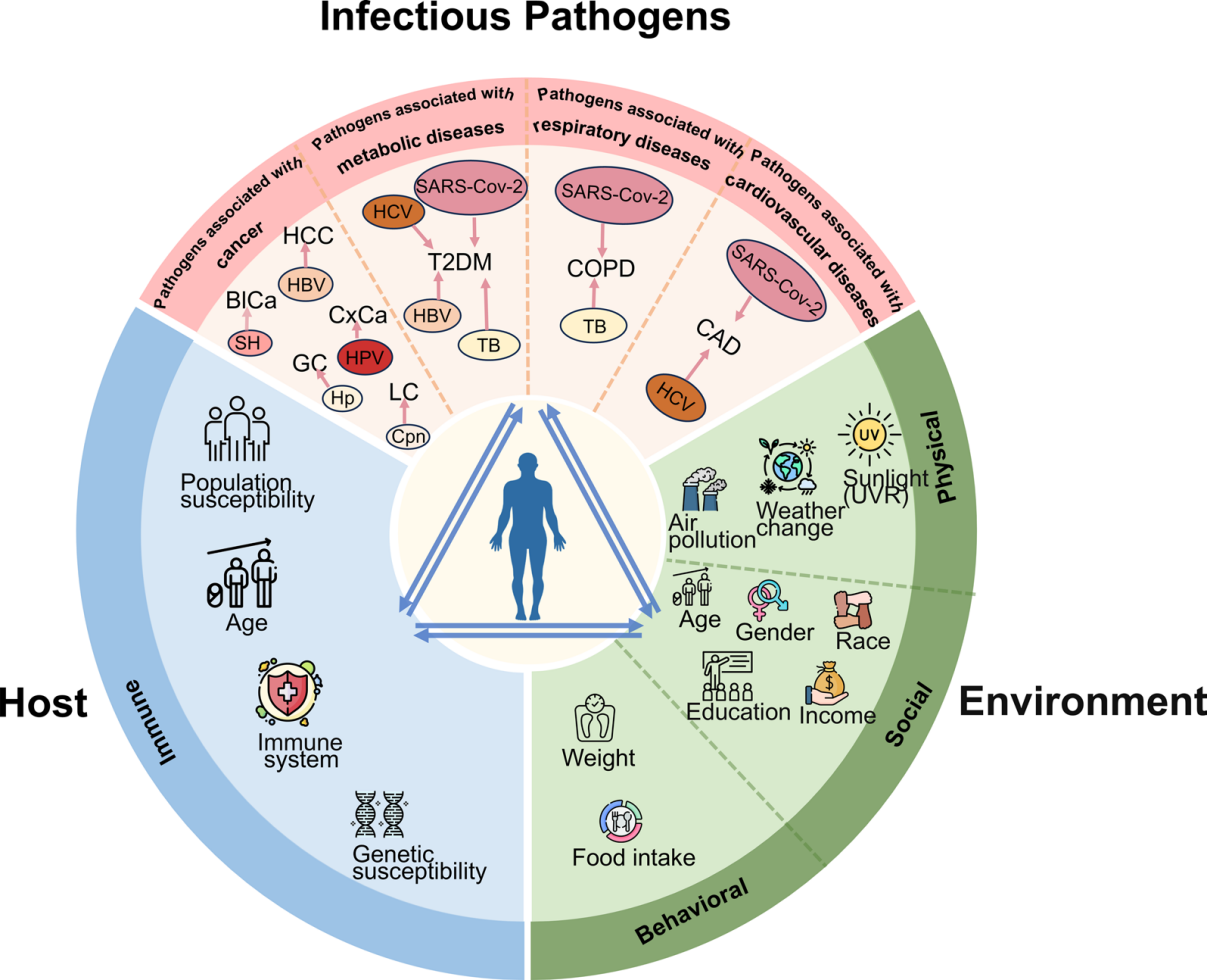
The interaction between pathogen, host and environment presented as the Triangle Model. The broad, inner circle represents the individual level and the outer the population level. *BlCa* bladder cancer; *CAD* coronary artery disease, *COPD* chronic obstructive pulmonary disease, *Cpn* Chlamydia pneumoniae, *CxCa* cervical cancer, *GC* gastric cancer, *HCC* hepatocellular carcinoma, *HBV* Hepatitis B virus, *HCV* hepatitis C virus, *Hp* Helicobacter pylori, *HPV* human papillomavirus, *LC* lung cancer, *SARS-CoV-2* severe acute respiratory syndrome coronavirus 2, *SH* Schistosoma haematobium, *TB* tuberculosis, *T2DM* type 2 diabetes mellitus, *UVR* ultraviolet radiation

The relationship between IDs and NCDs is further shaped by shared risk factors and overlapping biological, behavioural, and environmental determinants. As shown in Fig. 4, interactions among infectious pathogens, host characteristics, and environmental exposures contribute to the co-occurrence of IDs and NCDs. Pathogens can reach susceptible hosts through multiple routes, including direct or indirect contact, airborne transmission, and vector-borne spread [235]. Host vulnerability is determined by age, immune status, genetic predisposition, and underlying health status [235]. Many of the environmental exposures shown in Fig. 4 are established risk factors for NCDs [2]; through interactions with host susceptibility, these exposures may promote the onset or progression of chronic disease. In the presence of infectious pathogens, such interactions can give rise to comorbidity or multimorbidity involving IDs and NCDs, thereby increasing disease burden and complicating clinical and public health management.

Environmental factors affect not only host health and susceptibility to disease but also pathogen survival and transmissibility. These factors encompass biological, physical, and social determinants of health. Social determinants, including poverty, limited access to health care, and poor living conditions, can increase the burden of both IDs and NCDs [9]. Undernutrition, for example, is a well-established contributor to impaired immune function and increased susceptibility to IDs [236]. At the same time, poor-quality diets and overnutrition are recognised risk factors for NCDs, including cardiovascular disease and diabetes, through their contribution to obesity, metabolic dysfunction, and related cardiometabolic risk [237]. Thus, social and environmental conditions play a crucial role in influencing disease susceptibility and health outcomes [235]. In LMICs, where healthcare systems are often already constrained, this dual burden poses substantial clinical and public health challenges [238]. Populations in these settings frequently experience overlapping exposure to infectious risks and NCD determinants, making prevention and disease management particularly complex [238]. TB and T2DM, for instance, commonly coexist in these populations, with diabetes increasing the risk of TB disease, complicate treatment and clinical outcomes [179]. This overlap underscores the need for integrated health interventions that address IDs and NCDs simultaneously in resource-constrained settings.

### Impact on healthcare systems—policy and strategy implications

The dual burden of IDs and NCDs places substantial strain on healthcare systems, particularly in resource-limited regions [9]. During infectious disease outbreaks, such as the recent COVID-19 pandemic, healthcare resources are often redirected towards the acute response, disrupting routine care for chronic diseases [239]. Many patients with NCDs experienced interruptions in treatment, reduced access to medicines, and missed routine follow-up, potentially contributing to poorer chronic disease control [239]. This pattern highlights the urgency of building more resilient health systems and exposes structural limitations in current models of service delivery, financing, and resource allocation. Existing health policies and funding mechanisms often operate in a fragmented manner, addressing IDs and NCDs through separate programs and implementation strategies. Such fragmented resource allocation tends to constrain the overall effectiveness of interventions [240]. Funding agencies and policy makers should therefore reconsider existing financing frameworks, dismantle traditional funding silos, and establish cross-cutting mechanisms.

### Tailored, integrated intervention strategies

Recognizing the interconnectedness of IDs and NCDs provides a rationale for more integrated health strategies to address the dual burden [9]. Greater alignment of prevention, screening, and treatment across these disease domains could contribute to more sustained reductions in population-level disease burden. For example, vaccination programmes targeting infections such as HPV and HBV can reduce the incidence of infection-related cancers, whereas early detection and appropriate management of chronic diseases may help reduce infection-related complications in some populations [241]. Public health interventions should also address shared socioeconomic and environmental determinants that contribute to both IDs and NCDs, including sanitation, nutrition, and access to primary healthcare services [9]. Promoting healthy lifestyles—such as optimizing dietary patterns, increasing physical activity, and quitting smoking—not only reduces the risk of NCDs but also strengthens the immune system, making individuals more resilient to infections [242].

### Limitations

This Review has several limitations. First, because the associations between IDs and NCDs involve numerous disease categories and disease-pair combinations, no single search strategy could capture all potentially relevant links. Although we used broad searches to identify major disease pairings and then conducted targeted searches for specific combinations, some relevant associations might have been missed. Second, given the large number and substantial heterogeneity of disease pairings, pooled effect estimates were not calculated. Finally, for disease pairings with limited evidence from systematic reviews or meta-analyses, we included recent observational studies reporting relevant associations. However, evidence suggesting reverse causation or causal uncertainty might not have been fully captured. Causal interpretations of these associations should therefore be made with caution.

## Conclusion

IDs and NCDs can be interconnected through shared mechanisms, overlapping risk factors, and bidirectional pathways, thereby influencing disease onset, progression, and prognosis. Clinical practice should adopt integrated strategies that combine prevention, early detection, treatment, and long-term management to more effectively reduce the burden arising from the intersection of these two disease groups. At the public health level, the coexistence of infectious disease outbreak risks and the burden of chronic NCDs places increasing demands on health resource allocation, service delivery, and policy making. Health policies and financing mechanisms should move beyond disease-specific, siloed approaches towards systemic frameworks that support integrated prevention and control, coordinated management, and cross-sectoral action, to address the dual challenges of IDs and NCDs and ultimately improve population health.

## Supplementary Information

Supplementary Material 1

Supplementary Material 2

## Data Availability

No datasets were generated or analysed during the current study.

## Abbreviations

IDs: Infectious diseases
NCDs: Non-communicable diseases
WHO: World Health Organization
LMICs: Low-income and middle-income countries
BC: Before Christ
AD: Anno domini
HIV: Human immunodeficiency virus
AIDS: Acquired immunodeficiency syndrome
*CI*: Confidence interval
IARC: International agency for research on cancer
HPV: Human papillomavirus
HBV: Hepatitis B virus
HCV: Hepatitis C virus
EBV: Epstein-Barr virus
HTLV-1: Human T-cell leukemia virus type 1
HCC: Hepatocellular carcinoma
NPC: Nasopharyngeal carcinoma
SARS-CoV-2: Severe acute respiratory syndrome coronavirus 2
HBsAg: Hepatitis B surface antigen
OR: Odds ratio
NHL: Non-Hodgkin lymphoma
HDV: Hepatitis D virus
HEV: Hepatitis E virus
HL: Hodgkin lymphoma
RR: Risk ratio
GC: Gastric cancer
CC: Cervical cancer
STI: Sexually transmitted infection
OPC: Oropharyngeal carcinoma
LC: Larynx cancer
EGFR: Epidermal growth factor receptor
TGF-βR: Transforming growth factor beta receptor
AC: Anal cancer
DNA: Deoxyribonucleic acid
PeC: Penis carcinoma
VaC: Vagina carcinoma
HR: Hazard ratio
VC: Vulva carcinoma
KSHV: Kaposi sarcoma herpesvirus
KS: Kaposi sarcoma
NF-κB: Nuclear factor kappa B
PI3K/Akt: Phosphoinositide 3-kinase/protein kinase B
JAK/STAT: Janus kinase/signal transducer and activator of transcription
HLA-DQB1: Human leukocyte antigen DQB1
HLA-DRB1: Human leukocyte antigen DRB1
ATLL: Adult T-cell leukemia and lymphoma
ATL: Adult T-cell leukemia
HCMV: Human cytomegalovirus
ALL: Acute lymphoblastic leukemia
MCPyV: Merkel cell polyomavirus
MCC: Merkel cell carcinoma
LT: Large T antigen
BC: Bladder cancer
MDA: Mass drug administration
CRC: Colorectal cancer
CCA: Cholangiocarcinoma
CL: Cutaneous leishmaniasis
cSCC: Cutaneous squamous cell carcinoma
DALY: Disability-Adjusted Life Year
BCC: Basal cell carcinoma
VL: Visceral leishmaniasis
UI: Uncertainty interval
WASH: Water, sanitation, and hygiene
MD: Metabolic disease
T2DM: Type 2 diabetes mellitus
I^2^: Inconsistency index
TB: Tuberculosis
PTB: Pulmonary tuberculosis
RD: Respiratory disease
COPD: Chronic obstructive pulmonary disease
ACE2: Angiotensin-converting enzyme 2
ICU: Intensive care unit
BCG: Bacillus Calmette-Guérin
Flu: Influenza
NLRP3: NLR family pyrin domain-containing 3
CVDs: Cardiovascular diseases
CAD: Coronary artery disease
AMI: Acute myocardial infarction
STEMI: ST-segment elevation myocardial infarction
Cpn: Chlamydia pneumoniae
CxCa: Cervical cancer
Hp: Helicobacter pylori
SH: Schistosoma haematobium
UVR: Ultraviolet radiation
