# Supplementary material for: Co-managing the double burden: strategies for simultaneously tackling infectious and non-communicable diseases
Source: Infect Dis Poverty. 2026 Jul 31;15:85. doi: 10.1186/s40249-026-01477-y (PMC13425991; doi:10.1186/s40249-026-01477-y)
Supplement: Supplementary file 1 — Supplementary Material 1 [file 40249_2026_1477_MOESM1_ESM.docx]

**Table S1. Search strategy for the association between infectious pathogen infection and cancer development and progression**

| **PubMed** | |
| --- | --- |
| 1 | "Hepatitis B virus"[Title/Abstract] OR HBV[Title/Abstract] OR "Hepatitis C virus"[Title/Abstract] OR HCV[Title/Abstract] OR "Hepatitis D virus"[Title/Abstract] OR HDV[Title/Abstract] OR "Hepatitis E virus"[Title/Abstract] OR HEV[Title/Abstract] OR "Human papillomavirus"[Title/Abstract] OR HPV[Title/Abstract] OR "Epstein-Barr virus"[Title/Abstract] OR EBV[Title/Abstract] OR "Helicobacter pylori"[Title/Abstract] OR "Chlamydia pneumoniae"[Title/Abstract] OR Schistosoma[Title/Abstract] OR "Schistosoma haematobium"[Title/Abstract] OR "Schistosoma japonicum"[Title/Abstract] OR "Clonorchis sinensis"[Title/Abstract] OR "Opisthorchis viverrini"[Title/Abstract] OR "liver fluke*"[Title/Abstract] OR "human herpesvirus 8"[Title/Abstract] OR HHV-8[Title/Abstract] OR "Kaposi sarcoma herpesvirus"[Title/Abstract] OR KSHV[Title/Abstract] OR "HTLV-1"[Title/Abstract] OR "human T-cell leukemia virus type 1"[Title/Abstract] OR "Merkel cell polyomavirus"[Title/Abstract] OR MCPyV[Title/Abstract] OR "human cytomegalovirus"[Title/Abstract] OR HCMV[Title/Abstract] OR Leishmania[Title/Abstract] OR Cryptosporidium[Title/Abstract] |
| 2 | cancer*[Title/Abstract] OR neoplasm*[Title/Abstract] OR carcinoma*[Title/Abstract] OR malignan*[Title/Abstract] OR lymphoma[Title/Abstract] OR leukemia[Title/Abstract] OR "hepatocellular carcinoma"[Title/Abstract] OR "gastric cancer"[Title/Abstract] OR "cervical cancer"[Title/Abstract] OR "nasopharyngeal cancer"[Title/Abstract] OR "bladder cancer"[Title/Abstract] OR "colorectal cancer"[Title/Abstract] OR cholangiocarcinoma[Title/Abstract] OR "Kaposi sarcoma"[Title/Abstract] OR KS[Title/Abstract] |
| 3 | "odds ratio"[Title/Abstract] OR "relative risk"[Title/Abstract] OR "risk ratio"[Title/Abstract] OR "hazard ratio"[Title/Abstract] OR association*[Title/Abstract] OR risk*[Title/Abstract] OR link*[Title/Abstract] OR correlate*[Title/Abstract] |
| 4 | "systematic review"[Title/Abstract] OR "systematic reviews"[Title/Abstract] OR "meta-analysis"[Title/Abstract] OR "meta-analyses"[Title/Abstract] |
| 5 | #1 AND #2 AND #3 AND #4 |
| Limits | Free full text; English; humans; publication date from 2000/1/1–2026/4/20 |
| Results: 981 studies | |
| **Web of Science** | |
| 1 | ALL = ("Hepatitis B virus" OR HBV OR "Hepatitis C virus" OR HCV OR "Hepatitis D virus" OR HDV OR "Hepatitis E virus" OR HEV OR "Human papillomavirus" OR HPV OR "Epstein-Barr virus" OR EBV OR "Helicobacter pylori" OR "Chlamydia pneumoniae" OR Schistosoma OR "Schistosoma haematobium" OR "Schistosoma japonicum" OR "Clonorchis sinensis" OR "Opisthorchis viverrini" OR "liver fluke*" OR "human herpesvirus 8" OR HHV-8 OR "Kaposi sarcoma herpesvirus" OR KSHV OR HTLV-1 OR "human T-cell leukemia virus type 1" OR "Merkel cell polyomavirus" OR mcpba OR "human cytomegalovirus" OR HCMV OR Leishmania OR Cryptosporidium ) |
| 2 | ALL = (cancer* OR neoplasm* OR carcinoma* OR malignan* OR lymphoma OR leukemia OR "hepatocellular carcinoma" OR "gastric cancer" OR "cervical cancer" OR "nasopharyngeal cancer" OR "bladder cancer" OR "colorectal cancer" OR cholangiocarcinoma OR "Kaposi sarcoma" OR KS) |
| 3 | ALL = ("odds ratio" OR "relative risk" OR "risk ratio" OR "hazard ratio" OR association* OR risk* OR link* OR correlate*) |
| 4 | TI = ("systematic review" OR "systematic reviews" OR "meta-analysis" OR "meta-analyses") |
| 5 | #1 AND #2 AND #3 AND #4 |
| Limits | Publication years: 2000–2026; Language: English |
| Results: 1520 studies | |
| **Scopus** | |
| 1 | TITLE-ABS-KEY ( "Hepatitis B virus" OR HBV OR "Hepatitis C virus" OR HCV OR "Hepatitis D virus" OR HDV OR "Hepatitis E virus" OR HEV OR "Human papillomavirus" OR HPV OR "Epstein-Barr virus" OR EBV OR "Helicobacter pylori" OR "Chlamydia pneumoniae" OR Schistosoma OR "Schistosoma haematobium" OR "Schistosoma japonicum" OR "Clonorchis sinensis" OR "Opisthorchis viverrini" OR "liver fluke*" OR "human herpesvirus 8" OR HHV-8 OR "Kaposi sarcoma herpesvirus" OR KSHV OR HTLV-1 OR "human T-cell leukemia virus type 1" OR "Merkel cell polyomavirus" OR MCPyV OR "human cytomegalovirus" OR HCMV OR Leishmania OR Cryptosporidium ) |
| 2 | TITLE-ABS-KEY ( cancer* OR neoplasm* OR carcinoma* OR malignan* OR lymphoma OR leukemia OR "hepatocellular carcinoma" OR "gastric cancer" OR "cervical cancer" OR "nasopharyngeal cancer" OR "bladder cancer" OR "colorectal cancer" OR cholangiocarcinoma OR "Kaposi sarcoma" OR KS ) |
| 3 | TITLE-ABS-KEY ( "odds ratio" OR "relative risk" OR "risk ratio" OR "hazard ratio" OR association* OR risk* OR link* OR correlate* ) |
| 4 | TITLE-ABS-KEY ( "systematic review" OR "systematic reviews" OR "meta-analysis" OR "meta-analyses" ) |
| 5 | #1 AND #2 AND #3 AND #4 |
| Limits | Language: English; Subject area: Medicine; Document type: Review; Publication year: 2000–2026 |
| Results: 1959 studies | |

**Table S2. Search strategy for the association between non-communicable diseases and infectious disease susceptibility and clinical outcomes**

| **PubMed** | |
| --- | --- |
| 1 | "type 2 diabetes mellitus"[Title/Abstract] OR "type 2 diabetes"[Title/Abstract] OR T2DM[Title/Abstract] OR T2D[Title/Abstract] OR "chronic obstructive pulmonary disease"[Title/Abstract] OR COPD[Title/Abstract] OR "coronary artery disease"[Title/Abstract] OR "coronary heart disease"[Title/Abstract] OR CAD[Title/Abstract] OR CHD[Title/Abstract] OR "myocardial infarction"[Title/Abstract] OR "acute myocardial infarction"[Title/Abstract] OR AMI[Title/Abstract] |
| 2 | "COVID-19"[Title/Abstract] OR COVID19[Title/Abstract] OR "SARS-CoV-2"[Title/Abstract] OR "SARS CoV 2"[Title/Abstract] OR "severe acute respiratory syndrome coronavirus 2"[Title/Abstract] OR tuberculosis[Title/Abstract] OR TB[Title/Abstract] OR "Mycobacterium tuberculosis"[Title/Abstract] OR influenza[Title/Abstract] OR "human influenza"[Title/Abstract] OR flu[Title/Abstract] |
| 3 | "odds ratio"[Title/Abstract] OR "relative risk"[Title/Abstract] OR "risk ratio"[Title/Abstract] OR "hazard ratio"[Title/Abstract] OR association*[Title/Abstract] OR risk*[Title/Abstract] OR link*[Title/Abstract] OR correlate*[Title/Abstract] OR susceptib*[Title/Abstract] OR comorbid*[Title/Abstract] OR coinfect*[Title/Abstract] OR co-infect*[Title/Abstract] OR co-infection*[Title/Abstract] OR complication*[Title/Abstract] OR sever*[Title/Abstract] |
| 4 | "systematic review"[Title/Abstract] OR "systematic reviews"[Title/Abstract] OR "meta-analysis"[Title/Abstract] OR "meta-analyses"[Title/Abstract] |
| 5 | #1 AND #2 AND #3 AND #4 |
| Limits | Free full text; English; humans; publication date publication date from 2000/1/1–2026/4/20 |
| Results: 313 studies | |
| **Web of Science** | |
| 1 | ALL = ( "type 2 diabetes mellitus" OR "type 2 diabetes" OR T2DM OR T2D OR "chronic obstructive pulmonary disease" OR COPD OR "coronary artery disease" OR "coronary heart disease" OR CAD OR CHD OR "myocardial infarction" OR "acute myocardial infarction" OR AMI ) |
| 2 | ALL = ( "COVID-19" OR COVID19 OR "SARS-CoV-2" OR "SARS CoV 2" OR "severe acute respiratory syndrome coronavirus 2" OR tuberculosis OR TB OR "Mycobacterium tuberculosis" OR influenza OR "human influenza" OR flu ) |
| 3 | ALL = ( "odds ratio" OR "relative risk" OR "risk ratio" OR "hazard ratio" OR association* OR risk* OR link* OR correlate* OR susceptib* OR comorbid* OR coinfect* OR co-infect* OR co-infection* OR complication* OR sever* ) |
| 4 | TI = ( "systematic review" OR "systematic reviews" OR "meta-analysis" OR "meta-analyses" ) |
| 5 | #1 AND #2 AND #3 AND #4 |
| Limits | Publication years: 2000–2026; Language: English |
| Results: 548 studies | |
| **Scopus** | |
| 1 | TITLE-ABS-KEY ( "Diabetes Mellitus, Type 2" OR "type 2 diabetes" OR T2DM OR "Pulmonary Disease, Chronic Obstructive" OR COPD OR "chronic obstructive pulmonary disease" OR "Coronary Artery Disease" OR "coronary artery disease" OR CAD OR "Myocardial Infarction" OR "acute myocardial infarction" OR AMI ) |
| 2 | TITLE-ABS-KEY ( "COVID-19" OR COVID-19 OR SARS-CoV-2 OR "Tuberculosis" OR tuberculosis OR TB OR "human influenza" OR influenza OR flu ) |
| 3 | TITLE-ABS-KEY ( susceptibe OR risk OR associate OR comorbid OR coinfection OR "co-infection" OR complication OR severity ) |
| 4 | TITLE-ABS-KEY ( "systematic review" OR "systematic reviews" OR "meta-analysis" OR "meta-analyses" ) |
| 5 | #1 AND #2 AND #3 AND #4 |
| Limits | Language: English; Subject area: Medicine; Document type: Review; Publication year: 2000–2026 |
| Results: 478 studies | |
