# Supplementary material for: Co-managing the double burden: strategies for simultaneously tackling infectious and non-communicable diseases
Source: Infect Dis Poverty. 2026 Jul 31;15:85. doi: 10.1186/s40249-026-01477-y (PMC13425991; doi:10.1186/s40249-026-01477-y)
Supplement: Supplementary file 2 — Supplementary Material 2 [file 40249_2026_1477_MOESM2_ESM.docx]

**Table. Basic characteristics of the included studies**

| **Number** | **Authors** | **Year** | **DOI** | **Title** | **Country/Region** | **Surveytime** | **Disease** | **Number of participants** |
| --- | --- | --- | --- | --- | --- | --- | --- | --- |
| 1 | Mbaga, D. S et al | 2022 | 10.1371/journal.pone.0262903 | Hepatitis B, C and D virus infections and risk of hepatocellular carcinoma in Africa: A meta-analysis including sensitivity analyses for studies comparable for confounders | Africa | Inception-March 2021 | Hepatitis B Virus (HBV)/Hepatitis C Virus (HCV)/Hepatitis D Virus (HDV)+ Hepatocellular carcinoma (HCC) | Unclear |
| 2 | Zhu, X et al | 2019 | 10.1097/md.0000000000014755 | Hepatitis C virus infection is a risk factor for non-Hodgkin lymphoma: A MOOSE-compliant meta-analysis | No geographical restriction | Inception-October 2017 | Hepatitis C Virus (HCV) + B-cell non-Hodgkin lymphoma (B-NHL) | Unclear |
| 3 | Alfaiate, D et al | 2020 | 10.1016/j.jhep.2020.02.030 | Chronic hepatitis D and hepatocellular carcinoma: A systematic review and meta-analysis of observational studies | No geographical restriction | Inception-April 2019 | Hepatitis D Virus (HDV) + Hepatocellular carcinoma (HCC) | 98289 |
| 4 | Chang, T. E et al | 2022 | 10.1097/jcma.0000000000000606 | Hepatitis D virus dual infection increased the risk of hepatocellular carcinoma compared with hepatitis B virus mono infection: A meta-analysis | No geographical restriction | Inception-December 2019 | Hepatitis B Virus (HBV)/Hepatitis D Virus (HDV)+ Hepatocellular carcinoma (HCC) | Unclear |
| 5 | Yin, X et al | 2023 | 10.1016/j.canep.2023.102457 | Hepatitis E virus infection and risk of hepatocellular carcinoma: A systematic review and meta-analysis | No geographical restriction | Inception-May 2023 | Hepatitis E Virus (HEV) + Hepatocellular carcinoma (HCC) | 1297 |
| 6 | Hjalgrim, H.et al | 2005 | 10.1056/NEJMoa023141 | Characteristics of Hodgkin's lymphoma after infectious mononucleosis | Danish cohort/Swedish cohort | Unclear | Epstein-Barr virus (EBV) + Hodgkin lymphoma (HL) | More than 60000 young adults |
| 7 | Al-Khreisat, M. J et al | 2023 | 10.3390/diagnostics13122068 | Worldwide Prevalence of Epstein-Barr Virus in Patients with Burkitt Lymphoma: A Systematic Review and Meta-Analysis | No geographical restriction | Inception-January 2021 | Epstein-Barr virus (EBV)+ Burkitt lymphoma | Unclear |
| 8 | Liu, W. X et al | 2021 | 10.1186/s12935-021-01862-7 | The diagnostic value of EBV-DNA and EBV-related antibodies detection for nasopharyngeal carcinoma: a meta-analysis | Chain | Inception-January 2019 | Epstein-Barr virus (EBV) + Nasopharyngeal cancer (NPC) | 8382 patients，15089 individuals without NPC |
| 9 | Tavakoli, A et al | 2020 | 10.1186/s12885-020-07013-x | Association between Epstein-Barr virus infection and gastric cancer: a systematic review and meta-analysis | No geographical restriction | Inception-July 2019 | Epstein-Barr virus (EBV) + Gastric cancer (GC) | The pooled prevalence of EBV in 20361 gastric cancer patients |
| 10 | Wei, Feixue et al | 2024 | 10.1016/S0140-6736(24)01097-3 | Causal attribution of human papillomavirus genotypes to invasive cervical cancer worldwide: a systematic analysis of the global literature | No geographical restriction | December 2011–February 2024 | Human Papillomavirus (HPV) + Cervical cancer (CC) | 111902 cases，2755734 of normal cervical cytology |
| 11 | Rapado-González, Ó et al | 2020 | 10.3390/jcm9051305 | Association of salivary human papillomavirus infection and oral and oropharyngeal cancer: A meta-analysis | No geographical restriction | Inception-January 2020 | Human Papillomavirus (HPV) + Pharyngeal cancer (PC) | 2320 cases |
| 12 | Li, X et al | 2013 | 10.1093/infdis/jis698 | Human papillomavirus infection and laryngeal cancer risk: A systematic review and meta-analysis | No geographical restriction | Inception-May 2012 | Human Papillomavirus (HPV) + Laryngeal cancer (LC) | 2559 cases |
| 13 | Kreimer, A.R et al | 2015 | 10.1200/jco.2014.57.8435 | Human papillomavirus antibodies and future risk of anogenital cancer: a nested case-control study in the European prospective investigation into cancer and nutrition study | France, Germany, Greece, and Naples | January 1992–December 2000 | Human papillomavirus (HPV)+Anal cancer (AC) | 1829 patients |
| 14 | de Martel, C et al | 2017 | 10.1002/ijc.30716 | Worldwide burden of cancer attributable to HPV by site, country and HPV type | No geographical restriction | Unclear | Human papillomavirus (HPV)+ Penis carcinoma (PeC) | Unclear |
| 15 | Tung, H et al | 2024 | 10.1002/ijc.35105 | Human papillomavirus prevalence, genotype distribution, and prognostic factors of vaginal cancer | Taiwan, China | January 1989–December 2020 | Human papillomavirus (HPV)+ Vagina carcinoma (VaC) | 73 patients |
| 16 | Dolgasheva, D.S et al | 2025 | 10.15789/2220-7619-HPI-17789 | Human Papillomavirus in Vulvar Cancer: A Systematic Review | No geographical restriction | January 1993–December 2024 | Human Papillomavirus (HPV) + Vulvar cancer (VC) | Unclear |
| 17 | Kristen Stolka，et al | 2014 | 10.1016/j.canep.2014.02.006 | Risk factors for Kaposi's sarcoma among HIV-positive individuals in a case control study in Cameroon | Cameroon | January 2009–December 2011 | Kaposi sarcoma herpesvirus (KSHV) + Kaposi sarcoma (KS) | 175 cases, 1002 controls |
| 18 | Altieri, A et al | 2025 | 10.3390/v17101333 | HTLV-1 and ATLL: Epidemiology, Oncogenesis, and Opportunities for Community-Informed Research in the United States | No geographical restriction | Unclear | Human T-cell leukemia virus type 1 (HTLV-1) + Adult T-cell leukemia/lymphoma (ATLL) | Unclear |
| 19 | Francis, S.S et al | 2017 | 10.1182/blood-2016-07-723148 | In utero cytomegalovirus infection and development of childhood acute lymphoblastic leukemia | California | January 1995–December 1995 | Human cytomegalovirus (HCMV)+Childhood Acute Lymphoblastic Leukemia (ALL) | 268 cases，270 controls |
| 20 | Wijaya, W et al | 2022 | 10.3389/fonc.2022.868781 | Prevalence of Merkel Cell Polyomavirus in Normal and Lesional Skin: A Systematic Review and Meta-Analysis | No geographical restriction | Inception-August 2021 | Merkel cell polyomavirus (MCPyV) + Merkel cell carcinoma (MCC) | 5428 patients |
| 21 | Zheng, Y.L et al | 2012 | 10.1158/1055-9965.Epi-11-0589 | Urinary bladder cancer risk factors in Egypt: a multicenter case-control study | Egypt | July 2006–July 2010 | *Schistosoma haematobium (S. haematobium) + Bladder cancer (BC)* | 1886 cases，2716 controls |
| 22 | Almoghrabi, A et al | 2005 | 10.1179/136485905X19883 | A matched, case-control study of the association betweenSchistosoma japonicum and liver and colon cancers, inrural China | China | January 1995–December 2002 | *Schistosoma japonicum (S. japonicum) + Colorectal cancer (CRC)* | 142 cases，285 controls |
| 23 | Huang, Y.L et al | 2024 | 10.1016/j.actatropica.2024.107457 | The risk of hepatobiliary complications in Clonorchis and Opisthorchis infection: A systematic review and meta-analysis | No geographical restriction | Inception-December 2024 | *Clonorchis sinensis (C. sinensis) / Opisthorchis viverrini (O. viverrini) + Hepatobiliary complications (HCC)* | 34367 participants |
| 24 | Ouattassi, Naouar et al | 2022 | 10.1186/s43163-022-00335-6 | Squamous cell carcinoma associated with an active cutaneous leishmaniasis in immunocompetent patient: case presentation of an unlikely association and literature—review | No geographical restriction | Unclear | Cutaneous Leishmaniasis (CL) + Cutaneous squamous cell carcinoma (cSCC) | Unclear |
| 25 | Tumolskaya, N.I et al | 2022 | 10.18502/ijpa.v17i1.9031 | The first case of basal cell carcinoma in the scar fifty years after leishmanization | Russian Federation | January 2020–December 2020 | Cutaneous Leishmaniasis(CL) + Basal cell carcinoma（BCC） | 1 cases |
| 26 | Kalmi, G et al | 2020 | 10.1097/md.0000000000022787 | Visceral leishmaniasis in patients with lymphoma: case reports and review of the literature | Senegalese | January 2020–December 2020 | Visceral leishmaniasis (VL)+ Lymphoma | 2 cases |
| 27 | Abd El-Latif, N.F et al | 2023 | 10.31557/apjcp.2023.24.2.667 | Role of Cryptosporidium spp in development of colorectal cancer | Egypt | Unclear | *Cryptosporidium + Colon Cancer* | 100 patients，100 control |
| 28 | Gu,J et al | 2023 | 10.1080/14737159.2023.2277377 | A systematic review and meta-analysis on the relative and attributable risk of Helicobacter pylori infection and cardia and non-cardia gastric cancer | No geographical restriction | January 1990–December 2021 | *Helicobacter pylori (H. pylori) + Gastric cancer (GC)* | Unclear |
| 29 | Premachandra, N.M et al | 2022 | 10.1186/s13027-022-00425-3 | Chlamydia pneumoniae infections and development of lung cancer: systematic review | No geographical restriction | January 1997–December 2021 | *Chlamydia pneumoniae (C. pneumoniae) + Lung cancer (LC)* | Unclear |
| 30 | El-Naas, A et al | 2025 | 10.1080/22221751.2025.2492211 | New onset of type 1 and type 2 diabetes post-COVID-19 infection: a systematic review | No geographical restriction | Inception–October 2023 | COVID-19/SARS-CoV-2 + Type 2 Diabetes (T2DM); Diabetes (DM) | 8456639 |
| 31 | Zhang, J.L et al | 2025 | 10.1136/bmjopen-2024-090986 | Impact of COVID-19 infection on mortality, diabetic complications and haematological parameters in patients with diabetes mellitus: a systematic review and meta-analysis | No geographical restriction | 1 December 2019–14 January 2025 | COVID-19/SARS-CoV-2 + Diabetes (DM) | 1154674 |
| 32 | Zhou, J.Y et al | 2024 | 10.3389/fendo.2024.1429848 | Association of COVID-19 infection and the risk of new incident diabetes: a systematic review and meta-analysis | No geographical restriction | Inception–4 February 2024 | COVID-19/SARS-CoV-2 + Diabetes (DM) | Over 60 million |
| 33 | Fatoke, B et al | 2025 | 10.1186/s12879-025-11089-w | Type 2 diabetes mellitus as a predictor of severe outcomes in COVID-19-a systematic review and meta-analyses | No geographical restriction | 2019–march 2024 | COVID-19/SARS-CoV-2 + Type 2 Diabetes (T2DM); Diabetes (DM) | Unclear |
| 34 | McMurry, H.S et al | 2019 | 10.1002/dmrr.3066 | Coprevalence of type 2 diabetes mellitus and tuberculosis in low-income and middle-income countries: A systematic review | Low-income and Middle-income countries | 1990–2016 | Tuberculosis (TB) + Type 2 Diabetes (T2DM); Diabetes (DM) | Unclear |
| 35 | Alebel, A et al | 2019 | 10.1186/s12879-019-3892-8 | Prevalence of diabetes mellitus among tuberculosis patients in Sub-Saharan Africa: a systematic review and meta-analysis of observational studies | Sub-Saharan Africa | 10 August 2017–9 September 2017 | Tuberculosis (TB) + Diabetes (DM) | 13286 |
| 36 | Pitua, I et al | 2025 | 10.1186/s13098-025-01615-w | Diabetes and tuberculosis: a systematic review and meta-analyis of mendelian randomization evidence | No geographical restriction | Inception–8 October 2024 | Tuberculosis (TB) + Diabetes (DM) | Sample sizes ranged from 178671 to 424357 participants for PTB and 210865 to 433540 participants for DM |
| 37 | Hayashi, S et al | 2018 | 10.1111/tmi.13133 | Risk of active tuberculosis among people with diabetes mellitus: systematic review and meta-analysis | No geographical restriction | 1992–2017 | Tuberculosis (TB) + Diabetes (DM) | 22616623 |
| 38 | Reyes, F.M et al | 2021 | 10.3390/jcm10102087 | Assessment of the association of copd and asthma with in-hospital mortality in patients with covid-19. A systematic review, meta-analysis, and meta-regression analysis | No geographical restriction | Inception–12 June 2020 | COVID-19/SARS-CoV-2 + Chronic obstructive pulmonary disease (COPD) | 21309 patients (1465 with COPD and 633 with asthma) |
| 39 | Halpin, D.M.G et al | 2022 | 10.2147/jaa.s360985 | Epidemiology, Healthcare Resource Utilization , and Mortality of Asthma and COPD in COVID-19: A Systematic Literature Review and Meta-Analyses | No geographical restriction | January 2019–August 2021 | COVID-19/SARS-CoV-2 + Chronic obstructive pulmonary disease (COPD) | 115–211003 |
| 40 | Halpin, D.M.G et al | 2022 | 10.2147/jaa.s360985 | Epidemiology, Healthcare Resource Utilization , and Mortality of Asthma and COPD in COVID-19: A Systematic Literature Review and Meta-Analyses | No geographical restriction | January 2019–August 2021 | COVID-19/SARS-CoV-2 + Chronic obstructive pulmonary disease (COPD) | 115–211003 |
| 41 | Uruma, Y et al | 2022 | 10.1371/journal.pone.0276774 | Effect of asthma, COPD, and ACO on COVID-19: A systematic review and meta-analysis | No geographical restriction | Inception–27 September 2021 | COVID-19/SARS-CoV-2 + Chronic obstructive pulmonary disease (COPD) | 1229434 |
| 42 | Puri, A et al | 2022 | 10.1002/nop2.1126 | Comparison of comorbidities among severe and non-severe COVID-19 patients in Asian versus non-Asian populations: A systematic review and meta-analysis | Asia and non-Asia | Inception–24 March 2021 | COVID-19/SARS-CoV-2 + Chronic obstructive pulmonary disease (COPD) | Unclear |
| 43 | Feng, J.Y et al | 2025 | 10.3390/jcm14217639 | Bidirectional Association Between Tuberculosis and Chronic Obstructive Pulmonary Disease: A Systematic Review and Meta-Analysis | No geographical restriction | Inception–31 August 2025 | Tuberculosis (TB) + Chronic obstructive pulmonary disease (COPD) | Over 670000 |
| 44 | Merzah, M.A et al | 2023 | 10.1016/j.heliyon.2023.e19493 | A systematic review and meta-analysis on the prevalence and impact of coronary artery disease in hospitalized COVID-19 patients | No geographical restriction | 2019–2022 | COVID-19/SARS-CoV-2 + Coronary artery disease (CAD) | 357746 |
| 45 | Szarpak, L et al | 2022 | 10.3390/biology11020221 | Effect of Coronary Artery Disease on COVID-19-Prognosis and Risk Assessment: A Systematic Review and Meta-Analysis | No geographical restriction | Inception–2 November 2021 | COVID-19/SARS-CoV-2 + Coronary artery disease (CAD) | 49286 |
| 46 | Zuin, M et al | 2022 | 10.2459/jcm.0000000000001343 | Preexisting coronary artery disease among coronavirus disease 2019 patients: a systematic review and meta-analysis | No geographical restriction | Inception–8 December 2021 | COVID-19/SARS-CoV-2 + Coronary artery disease (CAD) | 27435 |
| 47 | Zhou, X et al | 2025 | 10.1016/j.virusres.2025.199594 | The association between influenza infection and acute myocardial infarction: A comprehensive systematic review and meta-analysis | No geographical restriction | Inception–20 February 2025 | Influenza + Acute Myocardial Infarction (AMI) | Unclear |
| 48 | Kawai, K et al | 2025 | 10.1161/jaha.125.042670 | Viral Infections and Risk of Cardiovascular Disease: Systematic Review and Meta-Analysis | No geographical restriction | Inception–July 2024 | Influenza + Acute Myocardial Infarction (AMI) | Unclear |
| 49 | Nguyen, T.Q et al | 2025 | 10.1093/cvr/cvaf092 | Systematic review and meta-analysis of respiratory viral triggers for acute myocardial infarction and stroke | No geographical restriction | Inception–26 August 2024 | Influenza + Acute Myocardial Infarction (AMI) | Unclear |
| 50 | Torabizadeh, C et al | 2023 | 10.30476/ijms.2022.93701.2504 | Prevalence of Cardiovascular Complications in Coronavirus Disease 2019 adult Patients: A Systematic Review and Meta-Analysis | No geographical restriction | December 2019–February 2021 | COVID-19/SARS-CoV-2 + Acute Myocardial Infarction (AMI) | 34379 |
| 51 | Thakker, R.A et al | 2022 | 10.1016/j.cpcardiol.2021.101032 | Comparison of Coronary Artery Involvement and Mortality in STEMI Patients With and Without SARS-CoV-2 During the COVID-19 Pandemic: A Systematic Review and Meta-Analysis | No geographical restriction | Unclear | COVID-19/SARS-CoV-2 + Acute Myocardial Infarction (AMI)/STEMI | 2266 |
| 52 | Zuin, M et al | 2023 | 10.1016/j.ijcard.2022.12.032 | Increased risk of acute myocardial infarction after COVID-19 recovery: A systematic review and meta-analysis | No geographical restriction | Inception–1 September 2022 | COVID-19/SARS-CoV-2 + Acute Myocardial Infarction (AMI) | 20875843 |
| 53 | Zhang, T et al | 2025 | 10.3389/fcvm.2025.1450470 | Cardiovascular outcomes in long COVID-19: a systematic review and meta-analysis | No geographical restriction | 1 December 2019–31 June 2022 | COVID-19/SARS-CoV-2 + Cardiovascular Disease | Unclear |
